# A spatial code for temporal information is necessary for efficient sensory learning

**DOI:** 10.1126/sciadv.adr6214

**Published:** 2025-01-08

**Authors:** Sophie Bagur, Jacques Bourg, Alexandre Kempf, Thibault Tarpin, Khalil Bergaoui, Yin Guo, Sebastian Ceballo, Joanna Schwenkgrub, Antonin Verdier, Jean Luc Puel, Jérôme Bourien, Brice Bathellier

**Affiliations:** ^1^ Université Paris Cité, Institut Pasteur, AP-HP, Inserm, Fondation Pour l’Audition, Institut de l’Audition, IHU reConnect, F-75012 Paris, France.; ^2^ Institut des Neurosciences de Montpellier, Université de Montpellier, INSERM, Montpellier, France.

## Abstract

The temporal structure of sensory inputs contains essential information for their interpretation. Sensory cortex represents these temporal cues through two codes: the temporal sequences of neuronal activity and the spatial patterns of neuronal firing rate. However, it is unknown which of these coexisting codes causally drives sensory decisions. To separate their contributions, we generated in the mouse auditory cortex optogenetically driven activity patterns differing exclusively along their temporal or spatial dimensions. Mice could rapidly learn to behaviorally discriminate spatial but not temporal patterns. Moreover, large-scale neuronal recordings across the auditory system revealed that the auditory cortex is the first region in which spatial patterns efficiently represent temporal cues on the timescale of several hundred milliseconds. This feature is shared by the deep layers of neural networks categorizing time-varying sounds. Therefore, the emergence of a spatial code for temporal sensory cues is a necessary condition to efficiently associate temporally structured stimuli with decisions.

## Teaser

The auditory system transforms temporal information in sounds into spatial information for rapid associations with decisions.

## INTRODUCTION

Many stimuli that drive selective behavioral decisions, such as phonemes and vocalizations (*1*–*3*), tactile textures and shapes (*4*, *5*), or the coherent motion of a moving animal (*6*), are identified based on the temporal structure of the sensory inputs. This can be directly experienced when listening to time-reversed renditions of common sounds such as words that are perceptually highly distinct from the original. In the brain, this temporal structure is associated with changes in when neurons fire, which neurons fire, and how much neurons fire. On the one hand, temporal stimuli drive neuronal activity sequences, described throughout the visual (*7*, *8*), auditory (*9*–*11*), tactile (*12*, *13*), and olfactory (*14*, *15*) systems, including sensory cortex. In single cortical neurons, these activity sequences carry information that is not available in the neuron’s mean firing rate (*4*, *7*, *9*, *13*, *15*). On the other hand, several studies have established that the time-averaged firing rate of many neurons is tuned to specific temporal cues, such as the speed or direction of motion in visual stimuli (*16*, *17*), the dynamics of tactile contacts (*12*, *18*), or amplitude and frequency modulations in sounds (*11*, *19*–*24*). This tuning generates a spatial code for the temporal structure of sensory inputs (*2*, *5*) based on the identity and firing rate of the set of activated neurons that is also referred to as a place or rate code. These spatial representations do not necessarily form anatomical maps [as in (*25*)] and can instead be widely distributed across a sensory area [as in (*2*)], but are referred to as “spatial” since they constitute a code that depends on the neuronal space rather than on time. Although there is evidence that temporal integration and spatial information increase throughout the sensory hierarchy (*11*), both temporal and spatial information coexist at all levels of sensory systems. Therefore, their respective functional importance is a long-standing question in sensory neuroscience. Correlative studies on this question yield conflicting results. Some results suggest that the spatial code contains sufficient information to explain performance in perceptual discrimination tasks (*26*). Other studies suggest that temporal codes contain information without which perceptual measures could not be explained (*27*, *28*).

To alleviate the intrinsic limits of correlative approaches, causal manipulations of neural activity patterns in space and time provide a way to directly test which code is actually exploited by downstream areas to drive perceptual decisions and behavioral output. Several optogenetic studies, using light-sculpting techniques, have demonstrated that, throughout sensory systems, spatial codes provide information that can drive discriminative behaviors (*29*–*31*). By contrast, temporal codes have so far only been causally implicated at the level of peripheral sensory networks (*32*, *33*)^,^. It is therefore unknown whether, at the cortical stage, temporal codes are also exploited by the downstream motor centers within which associations between stimuli and behavioral decisions are learnt (*34*).

To address this question, we focused on the auditory system because natural sounds contain rich temporal information (*35*) that is crucial for speech recognition (*1*) and for perceptual properties such as timber or even loudness (*36*, *37*). Accordingly, multiple studies have highlighted the ability of the auditory cortex (AC) to represent rich temporal motifs (*38*, *39*). We engineered optogenetically driven neural activity patterns in AC that could be distinguished exclusively based on either the spatial or temporal information they carry, focusing on the timescale of a few hundred milliseconds. Training mice to discriminate these patterns showed that learning mechanisms downstream to AC cannot efficiently use information contained either in temporal modulation of firing rate or in the relative timing between neural populations to drive decisions. However, we observed that sound representations are transformed between the subcortical and cortical stages such that temporal information about sounds is made available as a spatial code in AC that coexists with temporally structured responses at the few hundred milliseconds timescale. The emergence of this cortical spatial code explains previous accounts of the specific involvement of AC in learning to discriminate temporally structured sounds, since it bypasses the difficulty for downstream learning mechanisms to exploit temporally coded information. This transformation is also observed in deep neuronal networks performing sound categorization, suggesting that it is a general mechanism to associate temporally structured stimuli with perceptual decisions.

## RESULTS

### Patterned optogenetics allows engineering spatial and temporal codes

To causally evaluate if temporal information in the AC is used for learning behavioral associations with sounds, we aimed to design optogenetic activity patterns that can be discriminated based solely on their temporal structure. For this study, we selected examples of two distinct types of temporal information existing at the timescale of a few hundred milliseconds: (i) the temporal modulation of instantaneous firing rates within a neural population and (ii) the relative timing of activity in distinct neural populations. Following an earlier study (*40*), we reasoned that to specifically demonstrate the importance of temporal information, it is necessary to show behavioral discrimination between activity sequences that do not differ in the time-averaged spike rate per neuron.

**Fig. 1.**
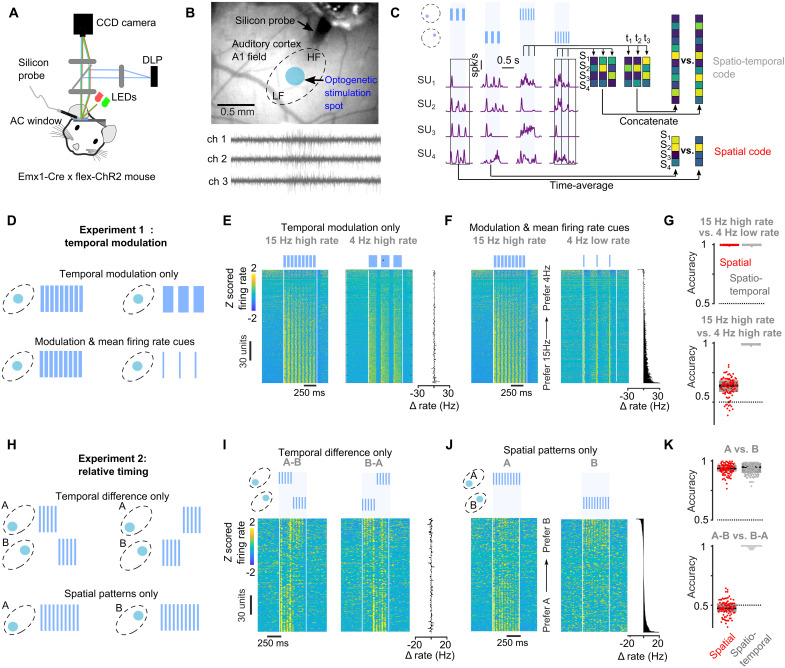
Parameterization of optogenetic stimulation to generate temporal and spatial neural patterns. (**A**) Sketch of experimental setup for simultaneous patterned optogenetic stimulation and single-unit recording in AC and for intrinsic imaging. (**B**) AC window showing the location of a stimulation spot along the tonotopic axis of the primary auditory field (A1) with 64-channel silicon probe inserted via a hole in the coverglass (top right) to record single-unit responses to light patterns and illustrative data from three channels. (**C**) Responses of four AC neurons to different optogenetic stimulation patterns illustrating how spatiotemporal and spatial codes are extracted. (**D**) Sketch of the temporal modulation patterns applied to a single spot on the AC. (**E** and **F**) *Z*-scored responses of 344 single units to the 15 Hz high rate versus and 4 Hz high rate (E) and 15 Hz high rate versus 4 Hz low rate stimulations (F) ordered by preference for 15-Hz versus 4-Hz stimulation. Right: Difference in each neuron’s average firing rate between stimulations. (**G**) Accuracy of a neural decoder trained to discriminate between the optogenetic patterns based only on spatial information or with spatiotemporal information (*n* = 344 units, bootstrap over units). (**H**) Sketch of the relative timing patterns applied to two spots A and B and the purely spatial pattern applied to either A or B. (**I** and **J**) *Z*-scored responses of 344 single units to A, B stimulations (I) and AB, BA stimulations (J), ordered by preference for A versus B stimulation. Right: Difference in each neuron’s average firing rate between stimulations. (**K**) Accuracy of a neural decoder trained to discriminate between the optogenetic patterns based only on spatial information or with spatiotemporal information (*n* = 344 units, bootstrap over units).

To generate such patterns, we delivered temporally modulated light spots with a video projector through a chronic cranial window positioned above the AC in awake Emx1-Cre × flex-ChR2 mice expressing channelrhodopsin-2 in a large population of pyramidal neurons (Fig. 1A) (*29*, *31*). The light spots targeted specific regions along the tonotopic axis of primary AC identified with intrinsic imaging (Fig. 1B and fig. S1A), and light intensity was calibrated to elicit similar firing rates levels to those naturally evoked by sounds (fig. S1B).

We first identified two periodic temporal modulation patterns of a single light spot that did not differ in their overall firing rate (Fig. 1D). We engineered these patterns by delivering light-on/light-off alternation patterns with systematically varying interpulse intervals and pulse durations while simultaneously recording single-unit activity using a silicon probe in awake passive mice (Fig. 1B). Periodic stimulation over 600 ms generated regularly timed responses in the cortex whose temporal structure could be easily identified on population raster plots (e.g., Fig. 1E). We found that combining short light-on pulses with short interpulse intervals could produce neural population firing rates similar to stimulations with longer light-on pulses and longer interpulse intervals (fig. S1, C and D). This provides the opportunity to generate different temporal sequences with very close overall spike counts. To quantify this accurately, we used population activity decoders, based on either the full spatiotemporal activity patterns chunked in 10-ms time bins (spatiotemporal decoder) or the time-averaged spatial activity patterns (spatial/firing rate decoder) (Fig. 1C and fig. S1E). This allowed us to select, among the few possibilities we identified, two stimulation patterns: 15 Hz high rate, ON: 49.8 ms—OFF: 16.6 ms versus 4 Hz high rate, ON: 149.4 ms—OFF 99.6 ms. Both patterns had very close average firing rates (fig. S1F) and could be easily identified by the spatiotemporal decoder but could barely be discriminated by the spatial/firing rate decoder (Fig. 1, E and G). As a control, we selected another combination based on the same stimulation periods but which produced very distinct firing rates (fig. S1F): 15 Hz high rate, ON: 49.8 ms—OFF: 16.6 ms versus 4 Hz low rate, ON: 16.6 ms—OFF: 233.2 ms pulse. These patterns could be easily discriminated by the spatial/firing rate decoder (Fig. 1, F and G).

We then used the same approach to construct two activity patterns that can only be discriminated based on the relative timing of neural activity across two stimulated regions A and B of the tonotopic map (Fig. 1H and fig. S1A). Each region was stimulated for 250 ms at 20 Hz (light ON: 25 ms, light OFF: 25 ms), and we contrasted A-B activity patterns in which A is played first and B afterward with the time symmetric B-A activity patterns. We chose an onset delay between A and B of 250 ms to avoid coincident firing in A and B. This is aimed at minimizing the synaptic interactions that are expected to occur (*32*, *41*) when two populations of a recurrent circuit like the cortex are active simultaneously (fig. S2). Such interactions can convert relative timing cues into spatial patterns of neuronal activity (fig. S2), an effect that we observed but that was of moderate magnitude for our stimulations (fig. S1H). We verified with electrophysiological measurements that A-B and B-A sequences generated time-symmetric activity patterns (Fig. 1I), which could not be discriminated based on time-averaged firing rates (Fig. 1K and fig. S1I). As a control, we used the spatial patterns alone (A or B spot), which, as we verified, generated distinct spatial patterns (Fig. 1J) and could be easily decoded based on time-averaged firing rates (Fig. 1K). Both pairs of patterns produced similar firing rate levels in AC (fig. S1I). Overall, our electrophysiological measurements established that all four pairs of patterns were efficiently discriminated by spatiotemporal decoders (Fig. 1, G and K), indicating that, theoretically, they should be equivalently discriminated if both spatial and temporal information are equivalently read out downstream of the AC.

### Spatial information is more efficiently learnt than temporal information

**Fig. 2.**
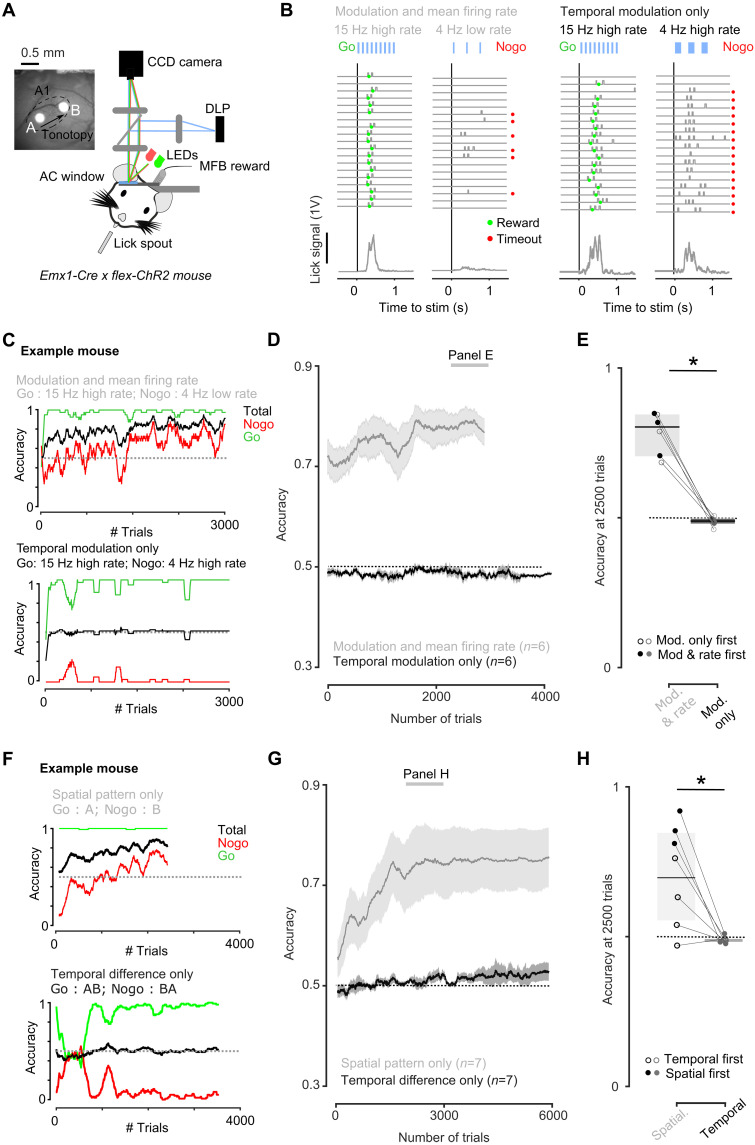
Sensory-motor learning is more efficient with spatial than temporal neural patterns. (**A**) Sketch of experimental setup for behavioral discrimination of patterned optogenetic stimulation in AC and cranial window from an example mouse showing the location of the stimulation spots in the tonotopic axis of the primary auditory field. (**B**) Sample lick traces (top) and mean lick signal (bottom) for Go and NoGo trials in the task with temporal modulation and firing rate cues that the mouse successfully learnt (left) and in the task with temporal modulation cues only in which the mouse failed to discriminate (right). (**C**) Learning curves for an example mouse performing the two tasks with temporal modulation. (**D**) Learning curves for all mice performing the tasks with temporal modulation (*n* = 7, error bars are SEM). (**E**) Accuracy at 2500 trials for all mice (paired Wilcoxon test, *P* = 0.031, signed rank value = 21, *n* = 6). (**F**) Learning curves for an example mouse performing the relative temporal order task and the spatial pattern task. (**G**) Learning curves for all mice performing each task (*n* = 7, error bars are SEM). (**H**) Accuracy at 2500 trials for all mice (paired Wilcoxon test, *P* = 0.032, signed rank value = 27, *n* = 7).

To evaluate how efficiently the firing rate, spatial, or temporal information isolated in our optogenetic patterns can be associated with behavioral decisions, we trained mice on four different Go/NoGo tasks, in which they had to discriminate one of the four pairs of patterns previously calibrated (Fig. 2, A and B). One group of mice was trained on the temporal modulation task and its control task in which firing rate also contributed information, i.e., patterns shown in Fig. 1, D to G (Fig. 2, C to E). Another group was trained on the relative timing task and its control spatial pattern task, i.e., patterns shown in Fig. 1, H to K (Fig. 2, F to H). For both groups, task order was counterbalanced across mice (fig. S3, A and B). All tasks consisted in licking within a 1.5-s opportunity window after the Go pattern onset to get a reward provided by medial forebrain bundle (MFB) stimulation, which leads to identical discrimination learning rates as water rewards in water-deprived animals (*42*). Licking for the NoGo pattern was punished by a timeout (Fig. 2B). We verified that mice were not responding to the light emitted by the stimulation device by masking the cranial window during behavior (fig. S3, C and D) and that they could readily detect both high and low firing rate patterns (fig. S3, E and F).

For firing rate differences (15 Hz high rate versus 4 Hz low rate), mice rapidly learned the discrimination reaching 70% accuracy within 69 ± 385 trials (Fig. 2, C to E). Consistent with previous results (*31*), the discrimination of spatial patterns (A versus B) was learnt within a larger but still relatively low number of trials (700 ± 938 trials to 70% accuracy; Fig. 2, F to H). By contrast, for the two tasks in which discrimination relied only on temporal information (15 Hz high rate versus 4 Hz high rate and A-B versus B-A), after 2500 training trials, none of the mice had learnt the task (Fig. 2, E and H), although the same mice could learn to discriminate the control spatial patterns. Mice trained first on temporal tasks and learnt more slowly the spatial tasks (Fig. 2H) likely due to the latent inhibition typically observed in conditioning (*43*). Pushing training to even higher trial numbers, up to 6000 for certain mice, only two of seven mice reached slightly above chance levels (~60%) for the relative timing discrimination and none for the temporal modulation discrimination (fig. S3, G and H). As a rough comparison, a previous study showed that with the same behavioral protocol, learning sound discrimination, even when difficult, always required less than 2500 trials (*42*). Reaction times to the patterns differing only in temporal structure were sufficiently slow at the task beginning to allow mice to integrate temporal information and similar to those for the spatial patterns, showing that mice were not simply responding impulsively (fig. S3, I to L). Therefore, discrimination training for well-controlled optogenetic patterns indicates that contrary to spatial information, temporal information in the AC is difficult to learn by downstream sensory-motor association processes.

### A spatial code for slow temporal cues is implemented by the AC

**Fig. 3.**
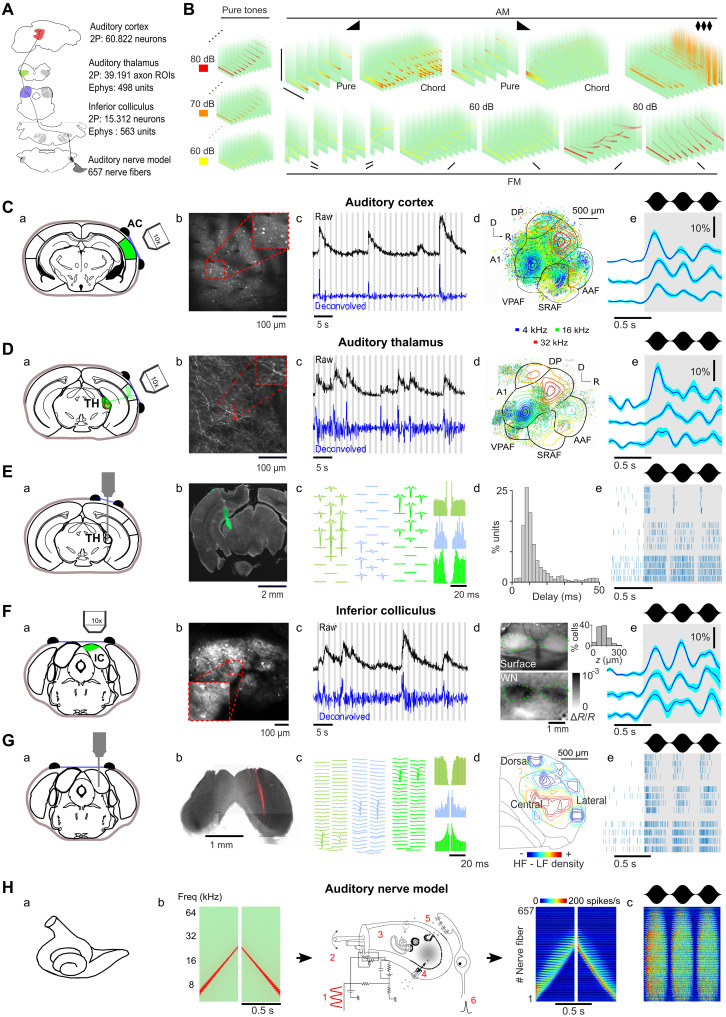
Extensive neural recordings throughout the auditory system. (**A**) Sketch of the auditory system and sample sizes at each level. (**B**) Spectrograms of the sound set. (**C**) (a) Schematic of imaging strategy, (b) sample field of view, and (c) raw (black) or deconvolved (blue) calcium traces (gray bar: sound presentation) for a sample neuron in AC. (d) Location of all recorded neurons, color-coded according to their preferred frequency at 60 dB, overlaid with the tonotopic gradients obtained from intrinsic imaging. (e) Response of three neurons to 3-Hz amplitude-modulated white noise. (**D**) Same as in (C) for thalamic axon imaging. (**E**) (a) Schematic of recording strategy in the TH, (b) sample histology with diI strained electrode track, (c) average waveforms and autocorrelograms of three single units, (d) response latencies of all single units, and (e) raster plot of five trials from three sample units in response to 3-Hz modulated white noise. (**F**) Same as (C) for dorsal IC except for (d) view of the cranial window and intrinsic imaging response to white noise. Inset histogram shows distribution recording depths. (**G**) Same as (E) for central IC, except for (iv) reconstruction of IC tonotopy from single units. (**H**) (a) Schematic of the cochlea and (b) of the biophysical model taking a sound as input and providing the responses of AN fibers. (c) Response to 3-Hz amplitude-modulated white noise. A1, primary AC; DP, dorsal posterior field; AAF, anterior auditory field; VPAF, ventral posterior auditory field; SRAF, suprarhinal auditory field.

Mice can learn discriminations of sounds that differ only in their temporal structure, such as time-symmetric frequency modulation (*44*) or different amplitude modulation frequencies (*26*, *27*). Since our results show that learning of temporal codes is difficult downstream of AC, the temporal information in sounds needs to be reformatted between the periphery and the cortex into spatial codes to be associated with behavioral decisions. This raises the question of where in the auditory hierarchy this reformatting occurs. Several studies have shown the existence of temporal integration over short and long timescales in the AC (*38*, *45*, *46*). These include spectrotemporal receptive field studies (*47*, *48*) and the wide range of studies demonstrating different forms of tuning for temporal cues in the firing rate activity of cortical or subcortical neurons in the auditory system (*2*, *23*, *24*, *49*, *50*), which provide building blocks for a spatial code for temporal cues. However, only a few studies have systematically compared AC to subcortical auditory centers and were limited to showing the temporal accuracy in AC for periodic amplitude modulations below 30 to 100 ms (*11*, *51*). We therefore lack a systematic evaluation of the precision of temporal and spatial codes across several auditory system regions for the longer timescales (>100 ms) at which they coexist and for the nonperiodic patterns evoked by natural sounds and covered by our optogenetic stimuli. Such a systematic evaluation is all the more necessary given that, even in AC, temporal codes at these timescales contain enough information to behaviorally discriminate temporal patterns in sounds, and it is still debated whether spatial information would be sufficient (*26*, *27*). We therefore performed large-scale recordings in awake mice in three successive regions of the auditory system: the inferior colliculus (IC), the auditory thalamus (TH), and the AC (Fig. 3A and table S1), combined with simulated auditory nerve (AN) responses. In each region, we measured the responses to a set of 140 sounds, mainly of 500-ms duration (full range: 100 to 1000 ms), which covered a large range of nonperiodic frequency and amplitude modulations, a few periodic amplitude modulations, and pure tones (Fig. 3B and table S2). In AC, we imaged 60.822 regions of interest (ROIs) throughout all subregions of the AC down to layer V (Fig. 3C and fig. S4, A to F). Calcium signals were linearly deconvolved (*21*), providing a temporal resolution of ~150 ms sufficient to follow slow temporal patterns produced by our 500-ms sounds (Fig. 3, C, D, and F). We also imaged 39.191 TH axonal boutons spread throughout AC and recorded 498 single units directly in TH (Fig. 3, D and E, and fig. S4, G and H). In the IC, we used silicon probe electrophysiology to record 563 single units in the primary IC (central nucleus of IC) and two-photon calcium imaging to record 13.132 ROIs from the more superficial secondary IC (dorsal cortex of IC) (Fig. 3, F and G). To provide insights into the structure of information entering the auditory system, we also integrate simulated sound responses from a detailed biophysical model of the cochlea calibrated against AN recordings (Fig. 3H and fig. S4, I and J) (*52*). Full details of the dataset are provided in the Supplementary Materials.

**Fig. 4.**
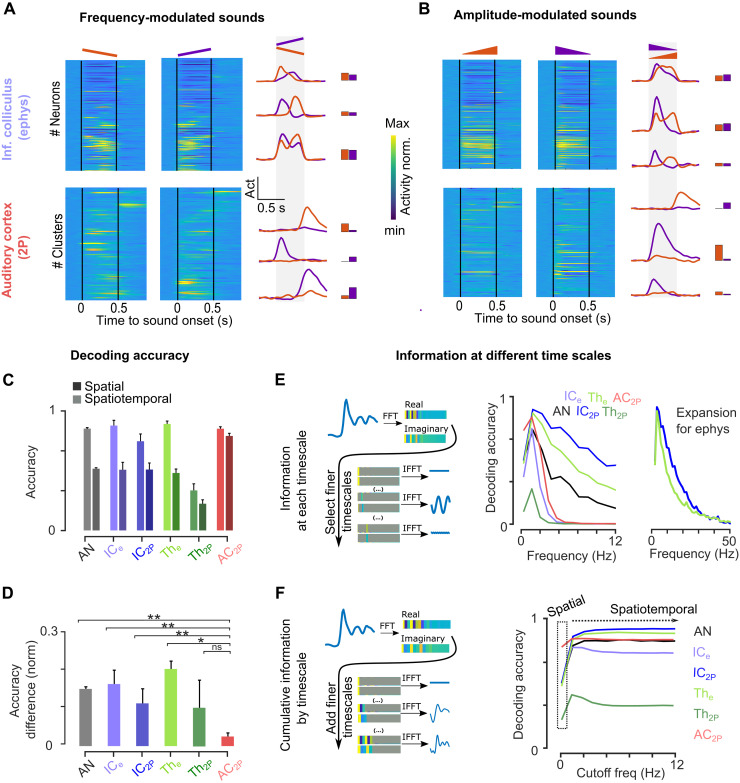
Increased accuracy of spatial coding in the AC. (**A** and **B**) Sample responses to up- and down-frequency sweeps (A) and up and down intensity ramps (B) from IC and AC neurons ordered by response amplitude. Example neurons are shown on the right. (**C** and **D**) Mean sound decoding accuracy for spatiotemporal and spatial codes in each area (C) and normalized difference between the two (D) (*P* value for 100 bootstraps, error bars are SD). (**E**) Left: Sketch illustrating the decomposition of population responses by timescale. Right: Mean decoding accuracy based on successive Fourier coefficients of neural responses. 0 Hz = spatial code. As expected, two-photon data only contained information up to 3 Hz, whereas electrophysiology data were informative even up to 30 Hz. (**F**) Left: Sketch illustrating the decomposition of population responses by timescale and the concatenation of successive Fourier coefficients to accumulate increasingly fine timescales. Right: Mean decoding accuracy based on cumulative Fourier coefficients of neural responses. Full statistics are reported in table S3.

To illustrate the observed transformation of sound representations from IC to AC, we plotted representative neural responses for pairs of time-reversed frequency sweeps (Fig. 4A) and intensity ramps (Fig. 4B), revealing a striking difference between the two structures. In the IC, neurons that respond to one of these time-varying sounds also respond to its time-reversed rendition with a similar firing rate level. Consistent with previous reports for frequency sweeps (*49*), peak amplitudes could vary across two time-reversed renditions (Fig. 4, A and B) and the temporal response profiles were roughly but not exactly symmetric. This indicates a sensitivity to modulation direction, which, however, appeared qualitatively weak when considering time-averaged firing rate levels (Fig. 4, A and B). By contrast, in the AC, we observed, along with neurons responding similarly to the IC, numerous neurons that evidently responded to one direction of modulation in a precisely timed manner but did not respond to the opposite direction. These qualitative observations suggest that the direction of modulation is coded in the AC both by the identity of the neurons that fire and by the time at which they fire, while in the IC, only the time at which neurons fire is fully informative.

To quantify this, we used the spatial and spatiotemporal population activity decoders previously defined (Fig. 1C) to predict which of our 140 sounds was played on any given single trial. While the spatiotemporal decoder performed equally well across auditory system stages, the accuracy of the spatial-only decoder strongly raised in the AC, drastically reducing the difference between the two decoders (Fig. 4, C and D). We verified that the differences between spatial and spatiotemporal decoders were not due to differences in sample sizes (fig. S5A). Moreover, to corroborate the visual observation that AC data are temporally structured (Fig. 4, A and B), we verified that the reduced difference between spatiotemporal and spatial decoders was not due to a lack of temporal precision in AC data. We did this by restricting the decoder analysis to specific timescale based on temporal Fourier decomposition of the data and by showing that information at the ~3-Hz timescale is sufficient for both calcium imaging and electrophysiology data to decode the slow temporal modulations of our sounds (Fig. 4, E and F). Therefore, population decoding validated our qualitative observations (Fig. 4, A and B) that the spatial code becomes much more informative in AC for temporal modulations over the timescale of about a few hundred milliseconds.

**Fig. 5.**
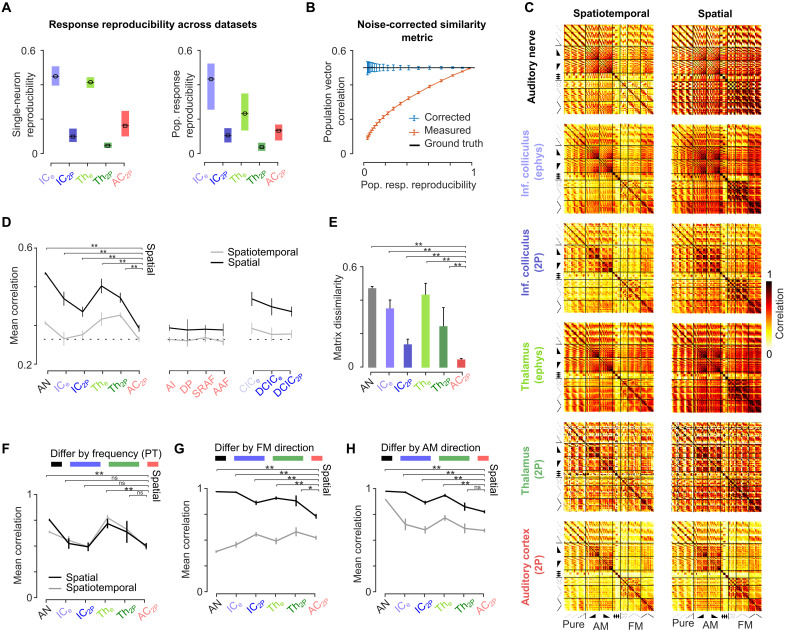
A spatial code for temporal cues emerges in the AC. (**A**) Reproducibility of single neuron (left) or population (right) responses measured as the mean intertrial correlation between responses across sounds (left: *n* = number of neurons per area, right: *n* = 140 sounds, error bars are quantiles). (**B**) Measured correlation of simulated data with 0.5 correlation to which different levels of noise were added before (orange) or after (blue) noise correction. (**C**) Noise-corrected RSA matrices for all sound pairs for temporal (left) or spatial (right) codes in each area of the auditory system. (**D**) Mean noise-corrected correlation for each auditory system area (*P* value for 100 bootstraps comparing rate correlation of each region to AC, error bars are bootstrapped SD). (**E**) Noise-corrected dissimilarity between RSA matrix structure of spatiotemporal and spatial codes (*P* value for 100 bootstraps, error bars are SD). [(F) to (H)] Mean noise-corrected correlation between sound pairs differing by only one acoustic property. (**F**) Pure tones at the same intensity differing by 0.33 octaves in frequency. (**G**) Frequency-modulated sweeps at same intensity and frequency differing by direction. (**H**) Amplitude ramps at the same frequency differing by direction. For sounds without temporal structure (F), the mean correlation of representations is similar in AC and IC. For time-symmetric sounds [(G) and (H)], all brain areas show larger spatial correlations than in the cortex, except for TH_2P_ in (H) likely due to the high variability of thalamic responses. Full statistics are reported in table S3.

The poor performance of our decoders on the thalamic axons dataset (Fig. 4C) indicated that the high levels of measurement noise (Fig. 5A) precludes reliable comparison with other datasets using decoding analysis that is sensitive to noise. To solve this issue and to verify that our conclusions were not affected by different noise levels across datasets, we quantified the differences between the neural representations of distinct sounds with a noise-independent metric. We used a numerically and analytically validated noise-corrected version of the Pearson correlation (*53*) (Fig. 5B; see the Supplementary Materials for mathematical derivations) to construct representational similarity analysis (RSA) matrices summarizing the similarity between spatial or spatiotemporal representations of any pair of sounds (Fig. 5C). The noise correction effectively compensated for the higher variability in thalamic axon data, judging from the similar correlation values across electrophysiological and imaging data in the thalamus (Fig. 5, C and D). RSA matrices clearly demonstrated that spatial and spatiotemporal representations become nearly identical in AC (Fig. 5C) as quantified by the significant drop in the dissimilarity across matrices when reaching AC (Fig. 5E). As suggested by the decoder analysis, this was due to an improvement of the spatial representations of all sounds that become more distinct in AC as shown by the significant drop in the average correlation between spatial representations of all pairs of sounds (mean of RSA matrix; Fig. 5D). It should be noted that the evolution of representations is not simply a monotonic process from cochlea to cortex. First, there is an initial drop in spatiotemporal correlation between the AN and the IC that does not show further decrease up to the cortex. Second, correlation increases in TH relative to IC before the strong decorrelation in AC. Finally, the dorsal IC that receives cortical feedback shows an intermediary profile, more similar to AC than central IC. There are therefore multiple processing steps in the auditory hierarchy before the strong decorrelation of spatial representations in AC. This improvement of spatial representations was specific to sounds with temporal modulation. This is illustrated by the significant drop in AC compared to AN, IC, and TH of the representation similarity between time-reversed sounds (Fig. 5, G and H), in line with our qualitative observations (Fig. 4, A and B), whereas representation similarity across pure tones of different frequencies was not improved in AC (Fig. 5F and fig. S5, C and D). We verified that these results were not due to sample size (fig. S5B).

Together, our systematic analysis demonstrates that temporal information in sounds at the few hundred milliseconds timescale becomes more efficiently encoded by spatial representations in AC than in AN, IC, and TH. This transformation happens without loss of temporal precision at this scale in the cortex (Fig. 4, E and F). AC therefore implements a hybrid code, with temporal and spatial representations that are both informative about the sound. The emergence of this hybrid code provides a solution to the inefficiency of temporal codes for learning behavioral associations suggested by our optogenetic experiments and strengthens the idea that spatial codes are necessary for efficient learning in natural settings.

### Inefficient learning with temporal codes explains AC’s involvement in temporal discrimination tasks

**Fig. 6.**
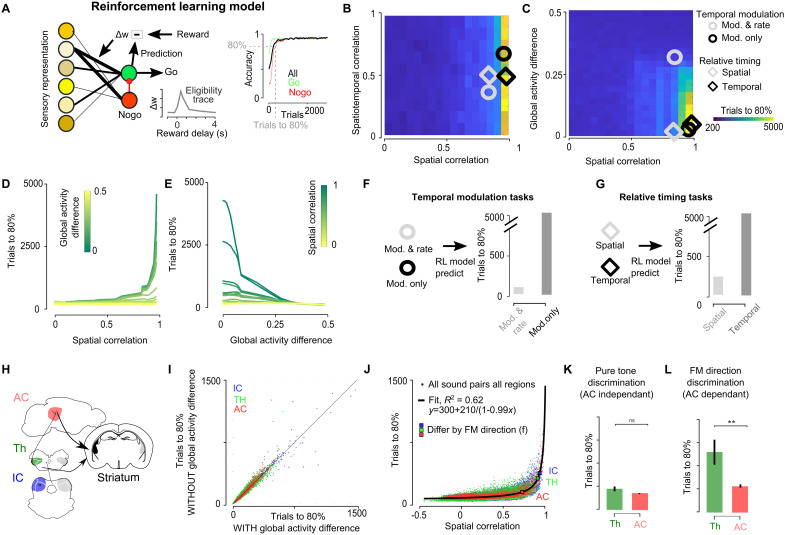
The spatial code determines learning speed and cortical involvement in an auditory Go/NoGo tasks model. (**A**) Left: Sketch of the reinforcement learning model and eligibility trace dynamics. Right: Example learning curve and the trial count to 80% accuracy. (**B** and **C**) Heatmap of the trial count to reach 80% accuracy at discriminating between a pair of sounds as a function of the spatiotemporal and spatial correlations (B) or as a function of their spatial correlation and global difference in activity level for simulated input representations. (**D** and **E**) Trial count to 80% accuracy as a function of the correlations of their spatial representations for different global activity differences (D) and vice versa. (**F** and **G**) Predicted number of trials to 80% accuracy for the two optogenetics tasks based on the spatial and spatiotemporal correlations and the global activity differences estimated from neural recordings. (**H**) Sketch showing the thalamic and cortical pathways for auditory learning. (**I**) Trial count to 80% accuracy for all pairs of sounds based on actual data (*x* axis) or on data in which the global activity difference between the two sounds is subtracted (*y* axis). (**J**) Trial count to 80% accuracy as a function of the correlations of their spatial representations for all sound pairs and all regions. Large squares show the mean correlation and learning time for time-symmetric frequency sweeps in IC, TH, and AC, and the black line shows the fit to data. (**K**) Predicted duration for learning a pure tone discrimination task based on thalamic (average of TH_e_ and TH_2P_) and cortical representations of sound pairs differing only by frequency (0.33 octave difference). (**L**) Predicted duration for learning to discriminate the two frequency sweep directions based on thalamic (average of TH_e_ and TH_2P_) and cortical representations of sound pairs differing only by the direction of the frequency sweep. Full statistics are reported in table S3.

If temporal codes are inefficient for learning sound discriminations (Fig. 2) and if temporal information in sounds becomes available in the format of a spatial code but only in AC (Figs. 4 and 5), a key function of AC may be to provide efficient representations to associate temporal information in sounds with behavioral decisions. This would provide an explanation for previous results showing that AC is necessary for rapidly discriminating time-varying sounds but dispensable for discriminating pure tones (*31*, *44*, *54*, *55*). To quantitatively verify if the hybrid spatial and temporal code emerging in AC is in line with this proposition, we used a learning model, blind to temporal information, and trained it to discriminate neural representations of sounds sampled in the auditory system. We slightly modified (see Materials and Methods) a previously developed minimal feedforward neural model, which accurately simulates mouse learning dynamics in Go/NoGo discrimination tasks (*56*) (Fig. 6A). This model learns to associate neural representations of sounds to decisions by reinforcing the synapses between sensory and motor levels based on a rate-based Hebbian synaptic learning rule that uses an eligibility trace to bridge the temporal gap between sound-driven activity and reward collection (*57*). In our model, Hebbian learning is driven by instantaneous firing rates pre- and postsynaptically and cannot potentiate synaptic connections based on the time structure of input activity.

To validate the model, we first evaluated if it reproduces our optogenetic experiments. We measured the number of trials needed for the model to discriminate artificially generated spatiotemporal input patterns that covered the three factors we varied in our optogenetic stimulations: difference in global firing rate levels (Fig. 1F) and differences in spatial (Fig. 1J) or temporal (Fig. 1, E and I) structure. As expected from the absence of time-sensitive learning rules, only differences in spatial pattern similarity or in global activity levels between the two discriminated inputs affected learning duration, both in a highly nonlinear manner (Fig. 6, B to E). Therefore, temporal sequences with strong spatial similarity and no difference in overall firing rates yield extremely inefficient learning. We measured pattern similarity and firing rate difference for the four pairs of optogenetic patterns used in our experiments (fig. S1, F, G, I, and J) and determined the learning duration predicted by the model (Fig. 6, F and G). Consistent with our behavioral data (Fig. 2), learning was particularly fast for discrimination of firing rates in a single stimulation spot (Fig. 6F), slightly slower for the discrimination of spatially distinct spots (Fig. 6G), and extremely slow for the two temporal discriminations (Fig. 6, F and G).

Given that decision areas involved in auditory discrimination tasks such as the striatum (*58*) or the amygdala (*59*) receive both cortical and subcortical inputs from the auditory system (Fig. 6H), we reasoned that differences in learning speed due to differences in sound representations across auditory system regions could explain why AC is required for certain discriminations and not for others. We therefore measured predicted learning duration for the pairs of sound representations we had recorded in AC, TH, or IC. To clarify the specific role of global firing rate differences and of spatial pattern similarity in biological representations, we first measured learning duration after equalizing global firing rates between each pair of sounds. As this had no impact on learning speed (Fig. 6I), we focused on pattern similarity and found, as for artificial patterns, a robust and steep nonlinear relationship between learning duration and noise-corrected measurements of spatial similarity (Fig. 6J). This nonlinear relationship provides a quantitative explanation for the causal involvement of AC in specific discrimination tasks. Simple sound pairs, such as pure tones differing enough in frequency (e.g., >0.33 octave), have low spatial representation similarity at all stages of the auditory system (e.g., correlation <0.75) (Fig. 5F). For this range of low correlation values, our model shows that learning occurs quickly and the impact of representation similarity on learning speed is marginal (Fig. 6J). Hence, the model predicts similar learning speeds whether it is based on thalamic or cortical representations (Fig. 6K), as observed for pure tone discriminations with intact or ablated AC (*31*). Contrariwise, sounds that differ only in their temporal structure, such as time-symmetric frequency modulations, have spatial representations that are highly correlated subcortically (>0.9) and clearly less in the cortex (0.74) (Fig. 5G). On the basis of these values, our model predicts a ~3-fold decrease in learning duration with cortical representations compared to thalamic representations (Fig. 6L). This is in line with the observation that pretraining AC ablation severely prolongs discrimination learning for time-reversed frequency sweeps (*54*). Also, if one postulates that learning speed determines which stage of the auditory system is recruited for solving a sound discrimination task, the relationship between spatial representation and learning duration in our model can explain the strong impact of posttraining AC inactivation on discrimination of temporal cues but not of pure tones (*31*, *44*, *55*).

### Spatial codes emerge from sound categorization in a deep neural network

**Fig. 7.**
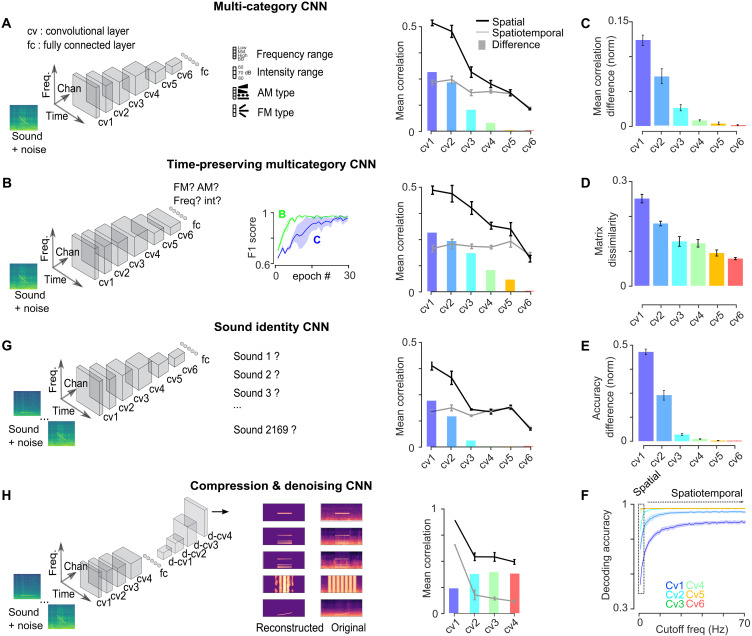
Categorization deep networks implement a spatial code for temporal cues in deeper layers. (**A**, **B**, **G**, and **H**) Left: Schematic of CNN architectures and target categories. Right: Mean response correlations for the spatial and spatiotemporal codes from RSA matrices constructed with the set of 140 sounds presented to mice (lines) and difference between the two codes (bars). (A) Multi-category CNN (*n* = 8 networks). (B) Multi-category CNN without shrinking of the temporal dimension (*n* = 8 networks). Inset shows learning curves from training epochs for networks in (A) and (B). (**C** to **F**) All graphs refer to the categorization CNN without temporal pooling and reproduce analysis shown in Figs. 4 and 5 for neural data. Error bars are SEM over trained networks. (C) Normalized difference between mean noise-corrected correlation for spatiotemporal and spatial codes. (D) Noise-corrected dissimilarity between RSA matrix structure of spatial and spatiotemporal codes. (E) Normalized difference between mean sound decoding accuracy for spatiotemporal and spatial codes. (F) Mean decoding accuracy based on cumulative Fourier coefficients of neural responses. (G) CNN (*n* = 8 networks) trained to identify each sound in noise. (H) Autoencoder CNN performing sound compression and denoising through a 20-unit bottleneck. cv, convolution block; d-cv, deconvolution block (see Materials and Methods for architecture details).

Our results indicate that spatial representations are important for associating time-varying sounds to categorical behavioral outputs. The emergence of a spatial code for temporal information in the cortex may also more generally reflect computations related to the resolution of common perceptual tasks such as stimulus identification and categorization. To explore this theoretically, we analyzed representations in convolutional neural networks (CNNs) trained for different sound processing tasks (Fig. 7). These networks rely on a cascade of linear/nonlinear computations known to be implemented via synaptic integration in auditory system neurons. They therefore provide a plausible phenomenological model for testing how task demands influence representations. A first network was trained at categorizing key features of the stimuli presented to our mice: the frequency and intensity range, and the type of frequency and amplitude modulations present in the sounds (Fig. 7A and fig. S6A). After training, but not before, this network generated a spatial code for temporal cues in its deep layers, as shown by the convergence of spatial and spatiotemporal similarity (Fig. 7A and fig. S6B). This was also observed in a previously published CNN trained to categorize words and musical styles (*60*) (fig. S6E). Like typical CNNs, these networks implemented pooling mechanisms that increase the size of sensory receptive fields and shrink the temporal and spatial dimensions in deeper layers (*61*). This raises the possibility that the convergence between spatiotemporal and spatial codes is simply the consequence of temporal resolution due to this temporal shrinking, which is imposed by the architecture of the network and not learnt because of the task. To rule this out, we trained a second CNN without pooling over the temporal dimension (Fig. 7B). We observed that even without temporal shrinking, the categorization CNN learns the task more slowly but also produces a spatial code for temporal cues (Fig. 7B). Therefore, the constraint of learning sound categories, imposed by the task, is sufficient for the network to learn the necessary computations to implement a spatial code for temporal cues without any tailored architecture constraint. The network without temporal shrinking produced representations that were similar in many respects to representations in the auditory system (compare Fig. 7, C to F, with Figs. 4, E and F, and 5, D and E). In particular, the spatial code for temporal cues in the deeper layers did not involve a decreased temporal resolution of the representations as in AC (compare Fig. 7F and fig. S6C with Fig. 4, E and F). Hence, the hybrid code (spatial and temporal) that we observed in AC may reflect the double need for the late auditory system to enable categorical decisions on temporally structured sounds while preserving enough temporal resolution for other purposes. Even fine-grained categories led to a spatial code for temporal cues in CNNs, as seen when training for single sound identification in noise (assigning one label per sound) (Fig. 7G). In contrast, when the task involved no categorical output, CNNs did not produce spatial codes for temporal cues. To exemplify this, we trained a CNN with an autoencoder architecture, to denoise and compress sound representations through a small bottleneck of only 20 units without assigning specific labels to sounds (fig. S6, F and G). In the bottleneck layer, this network also uses a representation without any explicit temporal representation since it integrates from all temporal nodes (fig. S6H). Despite this temporal integration, we did not observe the emergence of a spatial code for temporal cues in earlier layers (Fig. 7H). This corroborates the idea that spatial code for temporal cues is important for categorical decisions and does not systematically emerge from temporal integration processes.

We also compared the structure of RSA matrices derived from each of our CNNs and from the auditory system. We first observed that early layers of the auditory system have representations that largely differ from any of the CNNs (fig. S6I). This indicates that these CNNs poorly emulate computations in early auditory stages. However, we observed a higher similarity of the RSA matrices between the deeper layers of the CNNs and the later layers of the mouse auditory system (fig. S6I). The best overall match was obtained with the CNN performing the multi-category task, and the worst match was obtained with the autoencoder network (fig. S6, J and K). This together supports the view that spatial representations for temporal cues emerge in the cortex due to the computational constraints of classifying sounds into perceptual objects assigned with categorical outputs.

## DISCUSSION

Our results show that the elaboration of a spatial code for temporal sound cues is an important function of the sensory cortical network. This is in line with the proposed role of AC in the encoding of auditory objects (*62*) such as phonemes, vocalizations, or musical notes and previous observations of spatial representations for speech or natural sounds in the AC of humans and animals (*2*, *60*, *63*). However, our conclusions highlight that it is not the type of stimuli per se that determines whether cortical networks are leveraged but whether the representation of these stimuli requires conversion from spatiotemporal to spatial encoding to be discriminable.

A first necessary condition for an efficient spatial code is that single neurons respond with changes in average firing rate to particular temporal motifs. Such neurons tuned to the direction and speed of temporal modulation already exist subcortically in the IC and the TH (*11*, *23*, *24*, *48*, *51*). Thus, rate representations are already present subcortically at the single-neuron level but have been shown to be refined in the cortex. Cortical neurons have longer integration times and therefore show a loss of temporal resolution (*11*). This has recently been shown to be accompanied by an increase in sensitivity to slower and more complex temporal structures than subcortically (*64*). Although in this study we restricted our analysis to the 10- to 100-ms timescale, this suggests that the appearance of a rate code goes hand in hand with the detection of temporal patterns with slower timescales that are present in natural acoustic stimuli. A second necessary condition is that these specific responses are sufficiently diverse and complementary across neurons to cover all the information contained in the temporal dimension. While the first condition was extensively studied in the past, the second was never investigated. Our use of a noise-corrected population analysis tools allows addressing this question to demonstrate that the re-encoding of temporal motifs via a spatial code is only complete in the cortex.

Our results demonstrate that this spatial encoding is necessary for efficient learning of sensory-motor associations. This constraint may arise from the plasticity rules implemented in associative centers such as the striatum or the amygdala, which link sensory information to behavioral responses that have their own unrelated temporal structure. Our results suggest that the challenge of associating the distinct temporalities of sensory signals and motor responses is resolved via spatial representations for temporal cues. We expect that the knowledge of this constraint will be key to re-encode perception at the cortical level, e.g., in the context of the development of cortical implants, complementing previous approaches with electrical microstimulation that sought to establish criteria for driving perception (*65*).

Several computational models (*66*, *67*) and experimental findings (*68*, *69*) have identified plasticity mechanisms by which temporal sequences can be associated to a specific neuronal output. These are based on the combination of spike timing–dependent synaptic plasticity (STDP) and neuronal integration mechanisms, such as those proposed to underlie learning of behavioral sequences after temporal compression in the hippocampus (*70*). Several factors could explain why such mechanisms are not efficiently recruited downstream of the AC for sensory-motor learning. First, our optogenetic stimuli, like many sounds, evolve on timescales of hundreds of milliseconds that are not suited for the short timescales of STDP (*71*). Second, different models indicate that under irregular spike train statistics as observed in vivo, STDP rules behave as standard Hebbian rules (*67*, *72*, *73*). While temporally precise sound responses in AC were often reported under anesthesia, more irregular spike trains are observed in the awake state (*74*). Accordingly, our optogenetic stimulations were calibrated to yield realistic firing rates (fig. S1B) but not to generate high temporal precision below a few ten milliseconds. Further mechanisms have been proposed to adapt learning to long timescales. The eligibility trace mechanism, present in various brain areas including striatum, gates plasticity based on feedback delayed for several seconds (*75*). This long integration window averages out the precise timing of pre- and postsynaptic activity coincidences and does not in itself enable learning to discriminate precise temporal motifs. Cortical mechanisms such as the persistent network dynamics thought to underlie working memory should also allow exploiting temporal information over long timescales, likely by building spatial codes accounting for a long history of events (*76*). However, behavioral data indicate that these mechanisms require very long training times to be engaged in mice (*77*).

A previous study by Yang *et al.* showed that rats can discriminate between time intervals varying across a few milliseconds between two electrical stimulations at two different AC locations (*78*). The results likely reflect the conversion of fast temporal information into a spatial code that can occur via various synaptic interactions in the cortical network (fig. S2) (*79*). This study therefore does not contradict our findings, but rather confirms that the cortex can efficiently transform temporal information in local circuits at short timescales into a spatial code exploitable by downstream learning mechanisms. This suggests a separation of labor in which sensory cortical areas transform specific temporal information into spatial codes (Fig. 4) and generic subcortical plasticity mechanisms associate spatial representations to behavior (*34*, *59*). Within this framework, the key role of cortex in the transformation of temporal to spatial information is fully in line with causal experiments indicating that temporal cues in subcortical areas can be behaviorally detected (*32*, *33*). To further test how the cortex exploits this local fine temporal information and converts it to spatial patterns would require the use of single-cell resolution optogenetic techniques. However, currently, this does not allow activating enough neurons to directly drive learning of injected patterns (*80*).

These future avenues highlight the potential of behavioral experiments relying not on external sensory stimuli but on fully controlled internal neural stimulations for rigorous hypothesis testing. This approach enables the isolation of specific coding strategies in specific areas without being limited by the intrinsic properties of sensory stimuli or unknown processing in earlier regions. In our case, given that sounds generate both spatially and temporally coded information in the cortex, testing their respective roles was impossible without imposing stimulations isolating the two coding strategies. As another example of the fruitfulness of this approach, Yang and Zador compared temporal sensitivity between visual, auditory, and somato-sensory systems using cortical stimulation (*81*). This is the only way to make a fair comparison of coding ability between modalities without the biases of highly different sensory stimuli. Finally, internally manipulating reward, using MFB stimulation for example, can also refine hypothesis testing. It simplifies modeling by removing factors such as satiation and allows fine control of timing and intensity, as shown in a recent study using nucleus accumbens stimulation (*82*).

The complexity of the circuits required for the emergence of spatial codes for temporal cues is unknown. In sound categorization CNNs (Fig. 7), this transformation is based on a nonlinear cascade of temporally local computations that iteratively detect spectrotemporal features. This shows that a cascade of simple local circuit motifs with short integration windows is sufficient to make temporal information accessible through a spatial code in AC. This computational logic is also implemented by models of AC based on modulation filter banks, which hypothesize that neural responses can be accounted for by supposing that neurons are tuned to more or less complex spectrotemporal filters (*47*, *48*, *83*). Our study also revealed two intriguing aspects of sound information processing in the auditory system. First, contrary to what is observed in CNNs or typical spectrotemporal detection models (Fig. 7), representations from IC to AC are transformed nonmonotonically with more correlated representations in TH compared to AC and IC (Fig. 4).

This may reflect additional functional or anatomical constraints that are not taken into account by models and that will also need to be disentangled. Second, it is remarkable that neural temporal information is largely preserved in the AC despite the accessibility of temporal information through a spatial code (Fig. 4). This coexistence of temporal and spatial coding schemes could serve to combine object-like representations with an explicit representation of the temporal details that are also perceived together with the object. Such a hybrid coding scheme can therefore support both categorical behavioral outputs and the locking of precisely timed motor commands to sound features as, for example, in vocal optimization or vocal turn taking (*84*, *85*).

## MATERIALS AND METHODS

### Animals

All mice used for imaging and electrophysiology were 6 to 14 weeks old male and female C57Bl6J mice that had not undergone any other procedures. For optogenetic stimulation, we used Emx1-IRES-Cre (Jax #005628) crossed with Ai27 (Jax #012567) mice. Mice were group-housed (two to six per cage) before and after surgery, had ad libitum access to food and water and enrichment (running wheel, cotton bedding, and wooden logs), and were maintained on a 12-hour light-dark cycle in controlled humidity and temperature conditions (21° to 23°C, 45 to 55% humidity). All experiments were performed during the light phase. All experimental and surgical procedures were carried out in accordance with French Ethical Committees #59 and #89 (authorizations APAFIS#9714-2018011108392486 v2 and APAFIS#27040-2020090316536717 v1).

### Surgery

Mice were injected with buprenorphine (Vétergesic, 0.05 to 0.1 mg/kg) 30 min before surgery. Surgical procedures were carried out using either intraperitoneal ketamine (Ketasol) or medetomidine (Domitor), which was antagonized with atipamezole (Antisedan, Orion pharma) at the end of the surgery or 3% isoflurane delivered via a mask. After induction, mice were kept on a thermal blanket during the whole procedure and their eyes were protected with Ocrygel (TVM Lab). Lidocaine was injected under the skin of the skull 5 min before incision.

For calcium imaging, craniotomies of 3 mm (IC) or 5 mm (AC) were performed above the IC or the AC. Injections of 150 nl of AAV1.Syn.GCaMP6s.WPRE (Vector Core, Philadelphia, PA; 10^13^ viral particles per milliliter; used pure for TH and diluted 30× for AC and IC) were made at 30 nl/min with pulled glass pipettes at a depth of 500 μm and spaced every 500 μm to cover the large surface of the IC or AC. The craniotomy was sealed with a circular glass coverslip. The coverslip and head post were fixed to the skull using cyanolit glue and dental cement (Ortho-Jet, Lang).

For electrophysiology recordings, the skull above the IC or above the cortex dorsal to the TH was exposed for ulterior craniotomy. A well was formed around it using dental cement to retain saline solution during recordings, and the head post was fixed to the skull using cyanolit glue and dental cement. To protect the skull, the well was filled with a waterproof silicone elastomer (Kwikcast, WPI) that could be removed before recording. The head post was fixed to the skull using cyanolit glue and dental cement (Ortho-Jet, Lang).

For patterned optogenetic stimulation of the cortex, a cranial window was placed above the AC as for calcium imaging but without viral injection. For MFB stimulation, a bipolar stimulation electrode (60-μm-diameter twisted stainless steel, PlasticsOne) was implanted using stereotaxic coordinates (antero-posterior−1.4, medio-lateral +1.2, dorso-ventral +4.8). It was then fixed along with the headplate to the skull using dental cement (Ortho-Jet, Lang).

After surgery, mice received a subcutaneous injection of 30% glucose and metacam (1 mg/kg). Mice were subsequently housed for 1 week with metacam delivered via drinking water or dietgel (ClearH20). Mice were given 1 week to recover from surgery without any manipulation. Then, for 4 days before recording, mice were habituated to head restraint for increasing periods of time (30 min to 2 hours). For electrophysiological experiments, the day before recording, animals were briefly anesthetized using isoflurane anesthesia (2%) to perform craniotomy and durectomy for electrode descent.

### Two-photon calcium imaging in the awake mouse

Imaging was performed using a two-photon microscope (Femtonics, Budapest, Hungary) equipped with an 8-kHz resonant scanner combined with a pulsed laser (MaiTai-DS, SpectraPhysics, Santa Clara, CA) set at 900 nm. We used a 10× Olympus objective (XLPLN10XSVMP), which provided a field of view of up to 1 × 1 mm. For AC, a 1 × 1 mm field of view was used. For IC, the field of view was adjusted to the size of the structure (~0.5 × 0.5 mm). For thalamic axons, the field of view was reduced to 0.22 × 0.22 mm. Images were acquired at 31.5 Hz.

### Electrophysiology in the awake mouse

Electrophysiology was performed using Neuronexus probes (1 × 32 linear probe for IC and 4 × 8 comb for TH). For track reconstruction, the electrodes were dipped in diI, diO, or diD (Vybrant Multicolor Cell-Labeling Kit, Thermo Fisher Scientific) before recording and allowed to dry at least 15 min before insertion. Recordings were performed using warmed saline filling the cyanolit glue well and in contact with the reference electrode. After each recording, the well was amply flushed and then refilled with Kwickast. A maximum of three recordings were performed per site. Data were sampled at 20 kHz using an Intan RHD2000 amplifier board.

For recordings during optogenetic stimulation, a small hole was drilled in the coverglass and a 1 × 64 linear probe was inserted into the stimulated region. During these recordings, optogenetic stimuli and sounds were presented randomly. Recordings were performed using warmed saline filling the cyanolit glue well and with a reference electrode chronically implanted into the brain. After each recording, the well was amply flushed and then refilled with Kwickast. The animals used for optogenetic calibration were a separate group of naive from those performing the task. This calibration is based on a partial sampling of the full activated neural population, and sampling biases may have omitted residual spatial information.

### Sound delivery

Sounds were generated with Matlab (The Mathworks, Natick, MA) and were delivered at 192 kHz with a NI-PCI-6221 card (National Instruments) driven by the software Elphy (G. Sadoc, UNIC, France) and feeding an amplified free-field loudspeaker (SA1 and MF1-S, Tucker-Davis Technologies, Alachua, FL) positioned 15 to 20 cm from the mouse ear. Sound intensity was cosine-ramped over 10 ms at the onset and offset to avoid spectral splatter. The head fixed mouse was isolated from external noise sources by soundproof boxes (custom-made by Femtonics, Budapest, Hungary or Decibel France, Miribel, France) providing 30-dB attenuation above 1 kHz. Sounds were calibrated in intensity at the location of the mouse ear using a probe microphone (Bruel & Kjaer, type 4939-L-002). For two-photon calcium imaging, the resonant scanner generated a harmonic background noise at 8 kHz [intensity at the mouse ear, 45-dB sound pressure level (SPL)].

During a recording session, each of the 140 sounds (sketched in Fig. 3B) was presented 15 times in random order. To be compatible with two-photon image acquisition, sounds were presented in 120 blocks of 32 s each, interleaved by a 15-s pause in a 94-min protocol. The list of all sound parameters can be found in table S2.

### Intrinsic optical imaging recordings in anesthetized mice

Intrinsic imaging was performed to localize AC in mice under light isoflurane anesthesia (1% delivered with SomnoSuite, Kent Scientific) on a thermal blanket. Images were acquired at 20 Hz using a 50-mm objective (1.2 numerical aperture, NIKKOR, Nikon) with a charge-coupled device (CCD) camera (GC651MP, Smartek Vision) equipped with a 50-mm objective (Fujinon, HF50HA-1B, Fujifilm) through the cranial window implanted 1 to 2 weeks before the experiment (4-pixel binning, field of view between 3.7 × 2.8 mm or 164 × 124 pixels at 5.58 mm/pixel). Signals were obtained under 780-nm light-emitting diode (LED) illumination (M780D2, Thorlabs). Images of the vasculature over the same field of view were taken under 530-nm LED illumination (NSPG310B, Conrad). Sequences of short pure tones at 80-dB SPL were repeated for 2 s every 30 s with 10 trials per sound. Acquisition was triggered and synchronized using a custom-made GUI in MATLAB. For each sound, we computed baseline and response images, 3 s before and 3 s after sound onset, respectively. The change in light reflectance ∆*R*/*R* was calculated for each repetition of each sound frequency (4, 8, 16, and 32 kHz, white noise) as the difference between the baseline and response image and was then averaged across all repetitions of a given tone frequency. Response images were smoothed applying a two-dimensional (2D) Gaussian filter (SD = 3 pixels). AC activity appeared as regions with reduced light reflectance changing with frequency, revealing the tonotopic maps of its different subfields. To align intrinsic imaging responses across different animals, the 4-kHz response was used as a functional landmark. The spatial locations of maximal amplitude responses in the 4-kHz response map for the A1, A2, and AAF according to (*86*) (three points) were extracted for each mouse, and a Euclidean transformation matrix was calculated by minimizing the sum of squared deviations (RMSD) for the distance between the three landmarks across mice. This procedure yielded a matrix of rotation and translation for each mouse that was applied to compute intrinsic imaging responses averaged across a population of mice.

### Histology and immunostainings

To extract the brain for histology, mice were deeply anesthetized using a ketamine-medetomidine mixture and perfused intracardially with 4% buffered paraformaldehyde fixative. The brains were dissected and left in paraformaldehyde overnight and then sliced into 50-μm sections using a vibratome. Slices were either stained with cytochrome oxidase or directly mounted using a mounting medium with 4′,6-diamidino-2-phenylindole (DAPI). Analysis of the fluorescence band diI, diO, or diD allowed isolating up to three tracks per mouse for electrophysiological experiments.

For Vglut2 immunostainings, after fixation, tissues were rinsed in phosphate-buffered saline (PBS) and blocked in tris-buffered saline (TBS) supplemented with 5% (v/v) Normal Donkey Serum (Jackson ImmunoResearch) and 0.3% (w/v) Triton X-100. Then, sections were incubated for 48 hours at 4°C while rocking with a primary antibody: guinea pig anti-Vglut2 (1:500, Synaptic Systems #135404), followed by a 4-hour incubation with a secondary donkey anti–guinea pig immunoglobulin G (IgG) [F(ab′)_2_ fragments] (1:500, Jackson ImmunoResearch #706606148). Tissues were rinsed and mounted using ProLong diamond antifade (Life Technologies). Pictures of the brain sections were taken with an LSM 900 confocal microscope (Zeiss Microsystems) using 20× objective, whereas the magnified view of the thalamocortical boutons was obtained with Airyscan acquisition and 63× objective.

The labeled boutons (GCaMP alone in green; GCaMP with Vglut2 in yellow) were counted manually using ZEISS ZEN 2 microscope software in 12 sample regions selected within layer 1 AC in three different Airyscan images. The number of boutons was then calculated per volume tissue.

### Behavioral discrimination of patterned optogenetic stimuli

For patterned optogenetic activation in the mouse AC, we used a video projector (DLP LightCrafter, Texas Instruments) powered by a blue LED (center wavelength 460 nm). To project a 2D image onto the AC surface, the image of the micromirror chip was collimated through a 150-mm cylindrical lens (Thorlabs, diameter: 2 inches) and focused through a 50-mm objective (NIKKOR, Nikon). Light collected by the objective passes through a dichroic beam splitter (long pass, >640 nm, FF640-FDi01, Semrock) and is collected by a CCD camera (GC651MP, Smartek Vision) equipped with a 50-mm objective (Fujinon, HF50HA-1B, Fujifilm). To prevent visual perception of the optogenetic stimulations, a constant and strong background illumination provided by a white LED lamp was used and a cache was placed in front and close to the eyes to limit visual inputs.

The behavioral task aimed to teach mice to discriminate between two optogenetically induced patterns of activity in AC. The reinforcement used for the task used MFB stimulation in nondeprived mice. This protocol leads to similar learning speed, motor response timing, and psychometric measurements as water rewards in deprived animals.

#### Periodic temporal modulation task

In the “spatial discrimination protocol,” the two stimulations were composed of 600-ms illumination of a 300-μm-diameter spot placed at the center of AC presented with either 9× 48-ms pulses at 15 Hz or 3× 16.6-ms pulses at 4 Hz. In the “temporal discrimination protocol,” the two stimulations were composed of 600-ms illumination of a 300-μm-diameter spot placed at the center of the AC tonotopic axis presenting either 9× 49.8-ms pulses at 15 Hz or 3× 149.4-ms pulses at 4 Hz. Mice were trained on both tasks. Three mice were first trained on the temporal and then on the spatial task. Three mice were first trained on the spatial and then on the temporal task. In both versions of the task, the Go stimulus was always the 15-Hz stimulation, but the NoGo stimulus was switched between the two 4-Hz stimulations. This allowed a swift transfer between the two tasks without requiring animals to reverse the rule. Spot position was adjusted to avoid placing them above major vessels, which could lead to reduced illumination of neurons. Alignment of optogenetic stimulus locations across days was done using blood vessel patterns at the surface of the brain manually aligned to a reference blood vessel image taken at the beginning of the experiment.

#### Temporal order task

In the spatial discrimination protocol, the two stimulations were composed of 500-ms illumination of 300-μm-diameter spots placed at two different locations of AC. In the temporal discrimination protocol, the two stimulations were composed of a succession of two 250-ms illuminations of 300-μm-diameter spots at different locations in the cortex in one order (AB) or in the reversed order (BA). All light stimulations were temporally modulated at 20 Hz (25 ms ON, 25 ms OFF). Mice were trained on both tasks. Four mice were first trained on the temporal and then on the spatial task. Three mice were first trained on the spatial and then on the temporal task. The spots used in the task they first learnt were positioned at the two extremes of the tonotopic axis of A1. To minimize interference between the two subsequent tasks, the spots in the second task were positioned at two different locations, on the axis orthogonal to the tonotopic axis of A1 keeping the interspot distance equal. In both cases, spot position was adjusted to avoid placing them above major vessels, which could lead to reduced illumination of neurons (fig. S1, A and B). Alignment of optogenetic stimulus locations across days was done using blood vessel patterns at the surface of the brain manually aligned to a reference blood vessel image taken at the beginning of the experiment.

Behavioral experiments were monitored and controlled using a custom Matlab software controlling an input-output board (PCIe-6351, National Instruments), and the images were delivered by the video projector. Mice performed behavior for 1 hour per day. During the entire behavioral training period, food and water were available ad libitum as rewards were provided through the stimulation of the MFB.

MFB stimulation was delivered via a pulse train generator (PulsePal V2, Sanworks) that produced 2-ms biphasic pulses at 50 Hz for 100 ms at a voltage calibrated for each individual mouse to the minimal level that evoked sustained responding, using the protocol in (*42*). The stimulation was controlled with a solenoid valve (LVM10R1-6B-1-Q, SMC). A voltage of 5 V was applied through an electric circuit joining the lick tube and an aluminum foil on which the mouse was sitting. Lick events could be monitored by measuring the voltage across a series resistor in this circuit.

Training was broken down into three phases. (i) Lick training: On the first day, mice were presented with the lick tube and any licking was rewarded with immediate MFB stimulation. Mice generally began licking at high rates after 1 to 2 min, and the session was continued until mice reliably collected around 300 rewards. (ii) Go training: On the following day, Go trials were presented with 80% probability, while the remaining trials were blank trials (no stimulus). A trial consisted of a random intertrial interval (ITI: 0.5 to 1 s), a random “no lick” period (duration adjusted, see below), and a fixed response window of 1.5 s. The first lick occurring during the response window on a Go trial was scored as a “hit” and triggered immediate MFB stimulation. During initial go training, the no lick period was between 2 and 5 s to discourage nonspecific licking. When mice achieved >80% accuracy for the Go stimulus, a final Go session was performed during which a cache was placed over the window to verify that animals were not licking to remnant visual cues from the video projector (fig. S1, C and D). On this day and for subsequent Go/NoGo sessions, the no lick period was shortened to 1.5 to 3 s to obtain more trials per session. (iii) Go/NoGo training: After Go training, the second stimulus (NoGo) was introduced. During presentation of the NoGo sound, the absence of licking for the full response window was scored as a “correct rejection” (CR) and the next trial immediately followed. Any licking during NoGo trials was scored as a “false alarm” (FA), no stimulation was given, and the animal was punished with a random time-out period between 5 and 7 s. Each session contained 45% Go stimulations, 45% NoGo stimulations, and 10% blank stimulations. Note that the Go training was used to ensure high motivation of the animal during the Go/NoGo training by establishing an association between the optogenetic stimulus and the reward. For the time-independent task, this association was generalized to the NoGo stimulus, as seen through very high false alarm rates at the beginning of the Go/NoGo training (e.g., fig. S1B). This indicates that faster learning for the time-independent task is not due to an absence of generalization between the Go and NoGo stimulus when transitioning from the Go to the Go/NoGo training phases.

Learning curves were obtained by calculating the fraction of correct responses over blocks of 150 trials. Discrimination performance over one session was calculated as (hits + correct rejections)/total trials.

### Data preprocessing

For calcium imaging, ROIs corresponding to putative neurons (AC and IC) or axons and boutons (TH) were identified by using Autocell (*21*) (https://github.com/thomasdeneux/Autocell). Briefly, each frame of the recording was corrected for horizontal motion using rigid body registration. This step was visually controlled, and all sessions with visible *z* motion were eliminated. A hierarchical clustering algorithm, based on pixel covariance over time, agglomerated pixels up to a user-selected number of clusters corresponding to regions of the size of neurons of axons. Clusters were automatically filtered according to size and shape criteria. This step was controlled by a detailed visual inspection of selected ROIs during which ROIs without visually identifiable cell body shape were discarded.

For each ROI, the mean fluorescence signal *F*(*t*) was extracted together with the local neuropil signal *F*_np_(*t*). Then, 70% of the neuropil signal was subtracted from the neuron signal to limit neuropil contamination. Baseline fluorescence *F*_0_ was calculated with a sliding window computing the third percentile of a Gaussian-filtered trace over the imaging blocks. Fluorescence variations were then computed as *f*(*t*) = Δ*F*/*F* = [*F*(*t*) − *F*_0_]/*F*_0_. An estimate of firing rate variations *r*(*t*) was then obtained by linear temporal deconvolution of *f*(*t*): *r*(*t*) = *f*’(*t*) + *f*(*t*)/τ, *f*’(*t*) being the first derivative of *f*(*t*) and τ = 2 s the estimated decay of the GCAMP6s fluorescent transients. This simple method efficiently corrects the strong discrepancy between fluorescence and firing rate time courses due to the slow decay of spike-triggered calcium transients. It does not correct for the rise time of GCAMP6s, leading to remnant low-pass filtering of the firing rate estimate and a delay of ~100 ms between the firing rate peaks and the peaks of the deconvolved signal. Finally, response traces were smoothed with a Gaussian filter (σ = 31 ms).

Electrophysiological signals were high-pass filtered, and spike sorting was performed using the CortexLab suite (https://github.com/cortex-lab; UCL, London, England). Single-unit clusters were identified using kilosort 2.5 followed by manual corrections based on the interspike-interval histogram and the inspection of the spike waveform using Phy (https://github.com/cortex-lab/phy).

Both for imaging and electrophysiology data, single-trial sound responses were extracted (0.5 s before and 1 s after sound onset) and the average activity over the prestimulus period (0.5 to 0 s before sound onset) was subtracted for each trial.

### Reproducibility index and cell selection

To quantify the noise levels in the data, we calculated the mean intertrial correlation across all pairs of trials. The single-neuron reproducibility is then defined for each neuron as the average of the intertrial correlation for that neuron’s response to all 140 sounds. The population response reproducibility for each sound is defined as the average of the intertrial correlations of the full sequence of response of the whole neural population to that sound. ROIs or single units with reproducibility below 0.12 were classified as nonresponsive and were excluded from all analyses. As detailed in table S1, the number of responsive units and the corresponding fraction of the total number of units/ROIs recorded are as follows: AC, 19,414 (32%); TH, 3969 (12%); THE, 484 (97%), 5936 (39%), 442 (78%).

### Noise-corrected correlation

For each dataset, population representations were estimated after pooling all recording sessions in a virtual population. We used the correlation between population vectors as a metric of similarity between representations. The areas and techniques used to estimate neuronal ensemble representations yielded different levels of trial-to-trial variability due to intrinsic neuronal response variability and measurement noise. Most representation metrics are biased by variability, even after trial averaging, due to variability residues. For example, the correlation between two population representations (population vectors) will tend to decrease with respect to a variability-free estimate. When multiple observations of the same representations are available, it is possible to account for the impact of variability, by using specific estimators (*53*). Here, we showed analytically (see the Supplementary Materials) that the value of the Pearson correlation coefficient $\rho {\vec{\nu }}_{s}{\vec{\nu }}_{s\prime }$ between population vectors for two sounds ${\vec{\nu }}_{s}$ and ${\vec{\nu }}_{s\prime }$ in the absence of variability can be exactly estimated from noise-corrupted single-trial observations ${\vec{\nu }}_{s,r}$ and ${\vec{\nu }}_{s\prime ,r\prime }$ of ${\vec{\nu }}_{s}$ and ${\vec{\nu }}_{s\prime }$ when their dimension *N* approaches infinity, based on the following formula

$$\rho {\vec{\nu }}_{s}{\vec{\nu }}_{s\prime }\approx \frac{\frac{1}{R^{2}}\sum_{r,r\prime }\rho {\vec{\nu }}_{s,r}{\vec{\nu }}_{s\prime ,r\prime }}{\sqrt{\frac{1}{R^{2}{\left(1-R\right)}^{2}}\left(\sum_{r\neq r\prime }\rho {\vec{\nu }}_{s,r}{\vec{\nu }}_{s\prime ,r\prime }\right)\left(\sum_{r\neq r\prime }\rho {\vec{\nu }}_{s\prime ,r}{\vec{\nu }}_{s\prime ,r\prime }\right)}}$$

in which *r* and *r’* are single-trial indices and *R* is the total number of trials. This analytical result is confirmed by simulations for finite *N*, indicating that our estimator converges to the correlation value of the noise-free vectors (Fig. 5B). Code for calculating this estimator is provided with the online dataset. It is similar to the one previously proposed in (*87*).

Simulations for finite *N* show as expected that the estimator displays substantial deviations around the true correlation, which, however, average to zero. This leads to values of the estimator that can be outside [−1,1] in some cases. Our estimator displays extremely large deviations when $\frac{1}{R\left(R-1\right)}\sum_{r\neq r\prime }\rho {\vec{\nu }}_{s,r}{\vec{\nu }}_{s,r\prime }$ approaches 0, i.e., for representations that are dominated by noise. This occurred more often in datasets obtained by imaging, in particular in the thalamic axonal boutons dataset (TH). To limit imprecisions from these extreme values, we excluded from all datasets sounds for which $\frac{1}{R\left(R-1\right)}\sum_{r\neq r\prime }\rho {\vec{\nu }}_{s,r}{\vec{\nu }}_{s,r\prime }<0.01$ (retaining 101/140 sounds). In typical neural data, there are significant noise correlations across simultaneously recorded neurons within a trial. Therefore, the effective *N* can be much lower than the number of neurons. We minimized this contribution by shuffling trial identity for each neuron independently.

To evaluate the significance of mean correlation differences across all sound pairs for temporal and rate representations, we used a bootstrap procedure over the independently recorded sessions. This procedure had the advantage of providing a statistical assessment for biological replicability based on strictly independent measurements (neurons of the same recording are not fully independent statistically). The noise-corrected correlation measure was estimated 100 times after a random resampling of sessions with replacement. On the basis of this distribution, we measured the SD and calculated *P* values down to 0.01.

Spatiotemporal correlation was measured on vectors formed by concatenating the responses of all neurons throughout time (vector dimension = *N*_Neurons_ × *N*_TimeBins_). Rate correlation was measured first by time-averaging the responses of each neuron and then concatenating these values for all neurons (vector dimension = *N*_Neurons_). In both cases, we used data from the sound onset to 250 ms after the sound offset. To normalize the difference between temporal and rate correlation when comparing between areas, we use the following formula

$$\rho_{\text{diff}}=\frac{\rho_{t-\mathrm{av}}-\rho_{\text{seq}}}{1-0.5\left(\rho_{t-\mathrm{av}}-\rho_{\text{seq}}\right)}$$

### Population activity classifiers

To evaluate the accuracy of sound or optogenetic stimulation identification based on single-trial population responses, we trained a nearest-neighbor classifier on a subset of trials and cross-validated it on a distinct subset of trials. Training and testing sets were constructed by randomly selecting half of the trials for each unit. For each sound, we correlated the population response averaged over the training trials for this sound with the population response averaged over the testing trials for all the other sounds. The sound with the highest correlation was assigned as the prediction. Decoding accuracy is defined as the proportion of correctly assigned sounds.

Spatial and spatiotemporal were defined as for the correlation measures. Statistical significance was evaluated using the same bootstrap procedure as for the correlation measures. Decoding depends inherently on trial-to-trial noise, which limits the possibility of comparing between areas. This analysis serves to contrast spatial and spatiotemporal codes within an area.

To measure the information contained at different timescales, the temporal sequence of population activity was decomposed into its Fourier coefficients corresponding to a discrete set of timescales ranging from *T*, the 750-ms sound response duration, down to *2*∆*t,* where *∆t* is the discretization time of the dataset (1/2*∆t = f* the Nyquist frequency, *∆t* = *T*/24 *=* 31.25 ms for 2P-imaging data, and *∆t* = *T*/96 *=* 7.81 ms for electrophysiology data).

The Fourier coefficient *C_n,r_* for frequency *n*/*T* and neuron *r* is defined as

$$n,r=\sum_{k=1}^{2K}\nu_{r}\left(k\right)e^{\frac{i2\pi \mathrm{kn}}{\mathrm{Tf}}}$$

where ν*_r_*(*k*) is the activity of neuron *r* at timestep *k*, *i* = $\sqrt{-1}$, and *K* = *Tf*. Each coefficient is a complex number or, equivalently, a 2D vector. Hence, the activity sequence for a given neuron is represented by a vector of either 2 *K* data points or 2 *K* Fourier coefficients.

To measure the information present at a given timescale, we applied the population activity classifier on the population vector containing the 2 *N* Fourier coefficients for this timescale for the *N* neurons of the dataset (Fig. 4E). To measure information present above a particular timescale *T*_max_, we used the Fourier coefficients from 1 to *T*_max_ for each neuron and concatenated them into a 2*NT*_max_ population vector (Fig. 4F). Of note, when evaluating information at particular timescales, we did not apply any temporal filtering steps to avoid artifacts due to the finite size of the filter and preserve the full bandwidth of the data.

### Reinforcement learning model

We adjusted a previously published reinforcement learning model (*29*, *56*) to learn discriminations between pairs of temporal inputs. The model receives as inputs the temporal responses for two sounds: $\left[X_{\text{Go}}\left(t\right)\right]$ for the rewarded sound and $\left[X_{\text{NoGo}}\left(t\right)\right]$ for the nonrewarded sound. The model learns the synaptic weights between these input representations and a downstream decision circuit (Fig. 6B). This circuit is composed of a Go-unit, which outputs the decision (synaptic weights: $w_{E}$), and an inhibitory neuron that provides immediate linear inhibition to the reward neuron (synaptic weights: $w_{I}$). The temporal output, *y*(*t*), of the model can therefore be described as: $y\left(t\right)=\theta \left[w_{E}X\left(t\right)-w_{I}X\left(t\right)-\xi \right]$, where θ is the Heaviside step function and ξ is a time-independent Gaussian random noise process that models stochasticity of behavioral choices. The decision to go is made if the mean activity of the Go-unit within the response window 〈y(t)〉t is larger than 0.2 (〈.〉t denotes time averaging over 0.5 s).

The synaptic weights are updated according to a learning rule, which compares the reward prediction to the actual reward, assuming that reward prediction corresponds to the mean input received by the Go-unit. The learning rule has three particularities that have been previously shown to be important to account for mouse behavior (*56*) and compatible with our knowledge of synaptic plasticity rules. First, it is asymmetric: The learning rate is larger when an unexpected reward occurs than when an expected reward does not. Second, it is multiplicative: The learning rate at a given synapse depends on the current weight of that synapse. Finally, it takes into account the known dynamics of the eligibility trace in the striatum (*57*), which is a key target of both AC and TH in discrimination learning (*34*). The eligibility trace is a key mechanism in the “neo-hebbian framework” that aims to explain how synaptic plasticity can accommodate delays between action initiation and environmental feedback. This theory proposes that synapses that undergo pre-post coincidence before feedback are tagged via a long-lasting (about a few seconds) eligibility trace. Weight changes will only occur at these tagged synapses if they are subsequently exposed to neuromodulatory feedback before this eligibility trace decays. In line with this, in the striatum, potentiation of synapses is conditioned on dopamine release within a ~3-s time window following coincidence of pre- and postsynaptic activity. To implement this in our model, the temporal signal for the model input is convolved with a kernel corresponding to the temporal profile of dopaminergic plasticity gating taken from Yagishita *et al.* (*57*) before calculation of the weight update.

The learning rule is implemented as follows

$$\delta w_{E}=\lambda f\left[R-\sigma \left(w_{E}-w_{I}\right).X\right]\text{ElTr}$$

$$\delta w_{I}=-\lambda f\left[R-\sigma \left(w_{E}-w_{I}\right).X\right]\text{ElTr}$$

where λ is the learning rate, R is the action outcome (R=1 for reward, R=−1 for no reward), σ is the behavioral noise-level parameter that sets the model peak performance, and f() is the function that implements asymmetric learning such that

f(u)=u,if u<0

f(u)=νu,if u≥0

ν>1 is the learning rate asymmetry ratio

$$\text{ElTr}=\int_{0}^{T_{\text{ElTr}}}X\left(u\right)y\left(u\right)D\left(t-u\right)\mathrm{du}$$

where D(u) is the temporal function shown in Fig. 6A and taken from Yagishita *et al.* (*57*) and $T_{\text{ElTr}}=0.5\;s$.

To estimate the speed at which the model learns to discriminate between different patterns with independently varying spatial similarity, spatiotemporal similarity, and global activity differences, we used as input artificially generated vectors of population activity. Each artificial pattern was composed of 1000 neurons and 25 time bins. To generate these patterns, we generated an initial seed pattern by sampling each neuron’s activity at each time bin from a uniform distribution and then multiplying this by a random value that gave the overall activity level of the neuron. We then generated a second pattern with (i) different levels of spatiotemporal similarity by shuffling the order of time bins in varying numbers of neurons, (ii) different levels of spatial similarity by randomly sampling a new activity level in varying numbers of neurons, and (iii) different global activity levels by adding/subtracting a random value to all neurons in the pattern

We verified that this procedure created patterns in which the three variables varied independently. The model was then run for three independent simulations to average out the stochastic contribution, and we evaluated the number of trials to reach 80% based on the average learning curve over these three repeats. In Fig. 6, the global activity difference is defined as

$$\text{GAD}=\frac{\text{abs}\left(\vartheta_{1}-\vartheta_{2}\right)}{\vartheta_{1}+\vartheta_{2}}$$

This normalization allows us to compare results from the artificial data with those from the neural patterns recorded in response to optogenetic stimulations or to sound stimuli without the mean activity level impacting the result (since electrophysiological and calcium imaging data have different overall scales).

To estimate the speed at which the model learns to discriminate between different neural representations, we used as input the population vector time series for two different sounds from a given area. For calcium imaging, we first performed clustering of the response to reduce dimensionality. The model was then run for three independent simulations to average out the stochastic contribution, and we evaluated the number of trials to reach 80% based on the average learning curve over these three repeats. We observed in the data that spatial similarity and global activity difference for a given pair of sounds was strongly correlated. To disambiguate their contributions, we simulated learning with the model after having removed the average activity for each sound (WITHOUT global activity difference) while keeping the average activity different (WITH global activity difference).

For dimensionality reduction of the population vector, we performed agglomerative hierarchical clustering based on the Euclidean distance between each neuron’s full temporal response to all stimuli. The number of clusters was established by increasing the number of clusters until the sound-pair RSA matrix constructed from the clusters explained 95% of the variance of the matrix constructed from the full neural population. Clustering was performed independently for each dataset and yielded approximately 150 clusters in all areas. AC data displayed in Fig. 4 (A and B) represent clusters rather than single neurons.

### Convolutional neural networks

#### Augmented sound set

To train deep neural networks, we created an augmented sound set that covered all the basic parameters explored by the original set of 140 sounds used in experiments. We increased the number of sounds composing the sound set from 140 to 2169 by independently varying all features defining the sounds (frequency, intensity, amplitude modulation direction or period, frequency modulation direction, chord composition). Thereby, a given feature cannot be predicted based on other features as in the experimental sound set. We further augmented the sound set using the approach from (*60*). Each 500-ms sound is embedded at a random time in a randomly chosen 1.5-s snippet taken from an auditory scene (bus station, park, street…) with a random intensity (average: 53 dB, SD: 7 dB). We thus generated a total of 150,000 sounds for the test (6000), train (110,000), and validation (34,000) sets, respectively.

#### Task definitions

The multi-category task required the network to output a 14-element binary category vector in which 1 indicates that the sound presented belongs to 1 of 14 categories, divided into 4 groups within which categories are mutually exclusive: frequency range, intensity range, frequency modulation type, and amplitude modulation type. However, all sounds had to receive one label from each group. The group structure was not provided to the network, which therefore had to learn that a sound could not be simultaneously high and mid frequency for example. The categories were defined as follows:

1) Frequency range group: high frequency (4 to 8 kHz)/mid frequency (9 to 17 kHz)/low frequency 18 to 38 kHz)/broadband (white noise only). For chords and frequency-modulated chirps, the frequency value used for categorization was the average of all frequencies (i.e., middle of the chirp).

2) Intensity range group: high time-averaged intensity (80 dB)/mid time-averaged intensity (70 dB)/and low time-averaged intensity (60 dB). Amplitude-modulated sounds were assigned to their closest time-averaged range group. We obtained different overall intensities by ramping sounds sublinearly, linearly, or superlinearly.

3) Amplitude modulation group: Up-ramping/down-ramping/sinusoidal modulation/no modulation.

4) Frequency modulation group: Up chirp/Down chirp/no modulation.

The sound identification task required the network to output the identity of each of the 2169 different sounds without any category.

The convolutional autoencoder is a network trained to reproduce with minimal loss its input with the constraint of passing all information through a small central bottleneck layer. It is composed of an encoder subnetwork that processes the input to allow for compression in the bottleneck layer and a decoder subnetwork that reconstructs the output from the low-dimensional bottleneck representation.

#### Architecture definition and training

All networks take as input a 2D (time × frequency) matrix of the log-scaled spectrogram of the sound and must produce as output the labels described above. To achieve this, a series of convolutional blocks is applied to transform the input. All classification networks were built from a series of six blocks composed of the same layers:

1) convolution: the input is convolved by a filter whose weights the network must learn, and each layer applies multiple filters, generating a 3D matrix (time × frequency channel) from the initial 2D input (free parameters: kernel size, kernel stride, channel number)

2) activation: the output of the convolution is passed through a Relu nonlinear activation function

3) max pooling: the output of activation is downsampled by taking the maximal value of neighboring values (free parameters: pool size, pool stride)

4) dropout: to improve the robustness of training, during each training batch, a random 50% selection of connections are eliminated. During testing and validation, all connections are active

After these convolutional blocks, a final 64-node fully connected layer with a Relu nonlinearity allows to aggregate information across time, frequency, and channel dimensions. The output layer is obtained for the multilabel task by applying a sigmoid function to the fully connected output and for the identification task by applying a softmax function.

The output of the last layer allowed us to calculate the value of the loss function that comprises the error the network makes (categorical cross-entropy loss function) and an L1 regularization term to improve network robustness. This loss was then back-propagated during training to optimize the weights of the connections using the Adam optimizer.

Any given architecture requires arbitration across a wide range of free parameters, most notably the kernel and max pooling size and stride as well as the number of channels in each block. One approach to this problem is to perform a search across architectures to obtain optimal performance on the task. This has allowed optimization on ecologically relevant tasks to be proposed as a criterion for building deep networks that function like the brain. However, we focused on general properties of CNNs and were using a simple task without natural sounds. We therefore chose to assess the generality of our results on various architectures instead of performing an exhaustive search. We also verified the reliability of our results for a given architecture by using two different initialization weights per architecture. The four architectures we evaluated are defined as follows (CV, convolution layer; MP, max pooling layer; FC, fully connected layer; Ker, kernel size):

1) Input: 109 × 150; CV1: 109 × 150 × 18, Ker(3,3); MP; CV2: 55 × 75 × 20, Ker(5,5); CV3: 55 × 75 × 24, Ker(6,6); MP; CV4: 28 × 38 × 28, Ker(7,7); CV5: 28 × 38 × 32, Ker(8,8); MP; CV6: 14 × 19 × 32, Ker(9,9); FC: 64

2) Input: 109 × 150; CV1: 55 × 75 × 18, Ker(3,3); CV2: 55 × 75 × 20, Ker(5,5); CV3: 28 × 38 × 24, Ker(6,6); CV4: 28 × 38 × 28, Ker(7,7); CV5: 14 × 19 × 32, Ker(8,8); CV6: 14 × 19 × 32, Ker(9,9); FC: 64

3) Input: 109 × 150; CV1: 55 × 75 × 1, Ker(7,7)8; CV2: 55 × 75 × 20, Ker(7,7); CV3: 28 × 38 × 24, Ker(7,7); CV4: 28 × 38 × 28, Ker(7,7); CV5: 14 × 19 × 32, Ker(7,7); CV6: 14 × 19 × 32, Ker(7,7); FC: 64

4) Input: 109 × 150; CV1: 55 × 75 × 24, Ker(3,3); CV2: 55 × 75 × 24, Ker(5,5); CV3: 28 × 38 × 24, Ker(6,6); CV4: 28 × 38 × 24, Ker(7,7); CV5: 14 × 19 × 24, Ker(8,8); CV6: 14 × 19 × 24, Ker(9,9); FC: 64

One prominent consequence of the choice of CNN architecture is the way in which the input volume evolves throughout the network. Choosing a large stride in the convolutional or a large window size in the max pooling layer will lead to a shrinkage of the input dimensions (time and frequency). Given that the temporal dimension is preserved in the brain, we examined an architecture in which there is no shrinkage at all of the temporal dimension. To do this, we used the four same architectures described above, with the temporal dimension kept constant by setting all strides to 1 and eliminating max pooling. This results in a large expansion of the parameters in the network and affects training speed, although asymptotic performance remains the same (Fig. 7B).

The convolutional autoencoder receives as input the 2D spectrogram and must output a denoised spectrogram (spectrogram of the central sound without the background noise). The autoencoder was composed of four convolutional blocks as previously described in the encoding part and decoding networks, and the bottleneck is a fully connected, 20-node layer. Training was performed with an Adam optimizer, L1 and L2 regularization, and reconstruction error (mean squared error) as a loss function.

The CNN trained on word and musical genre recognition was previously published (*60*), and parameters have been made available at (https://github.com/mcdermottLab/kelletal2018). This network is composed of a central branch that splits into two branches, with one branch trained to identify musical genres and the other branch trained to identify words. In the original paper, the network was shown to achieve human-like performance and to qualitatively reproduce psychophysical measures during these tasks.

#### Analysis of CNN activations

Once the networks had been trained, we analyzed the responses of all nodes in each activation layer to the 140 sounds that were presented during experimental sessions. Each sound generates at a given layer a 3D matrix (time × frequency × channels). By considering the temporal response of each frequency × channel combination, we obtained analogs to the temporal response of individual neurons. We then applied the same analysis techniques to these artificial responses as described above for neural recordings. To perform decoding that requires multiple presentations of the same sound, we presented to the network multiple copies of each sound embedded in different noise backgrounds.

The bottleneck of the autoencoder has no structurally imposed temporal dimension, since all nodes receive input from all temporal bins. However, it was possible that the autoencoder “learnt” to implement such a temporal dimension by assigning specific nodes to specific time bins. To verify if this was the case, we presented a new set of sounds made up of very short pure tones to evaluate the temporal specificity of the bottleneck nodes (fig. S7G).

### Cochlear model

A computational model was implemented by adapting the seminal model of Meddis (*88*, *89*) to the mouse cochlea and validating it with mouse AN recordings (*52*). The model consists of a cascade of six stages recapitulating stapes velocity, basilar membrane velocity, inner hair cell (IHC) receptor potential, IHC presynaptic calcium currents, transmitter release events at the ribbon synapse, and firing response in AN fibers (ANFs) including refractory effects. The input model is a sound stimulus (in pascals). The output is a train of spiking events (in spikes/s) in 590 ANFs innervating 40 IHCs with a characteristic frequency (CF) distributed at regular intervals along the cochlear tonotopic from 5 to 50 kHz, 12 IHCs per octave. This distribution covered 82.8% of the basilar membrane length from 1.2% (apex) to 83.9% (base) in 2.07% increments. According to experimental data, the number of ANFs per IHC (*N*) was controlled by the relationship *N* = −0.0038*x*^2^ + 0.375*x* + 7.9, where *x* is the IHC location along the basilar membrane such that *x* = −56.5 + 82.5 log (CF), with *x* in percent from the apex and CF in kHz. By adjusting the time constant of the calcium clearance τ_Ca_ within each IHC synapse, ANFs with different spontaneous discharge rate (SR = 91.1 τ_Ca_^2.66^, with τ_Ca_ in ms and SR in spikes/s) were simulated from 0.5 to 95 spikes/s (21 ± 19.8 spikes/s, mean ± SD) to match the SR distribution reported in mouse AN.

### Fast timescale temporal to rate conversion in an excitation/inhibition model

We modeled in fig. S2 the response of two integrate-and-fire neurons connected by a single inhibitory synapse that receive inputs A (In_A_) and B (In_B_), respectively. These inputs represent the A- and B-driven populations in the optogenetics experiment of Fig. 1. The A and B inputs exactly reproduced our temporal-coded stimulation (225-ms duration, 20-Hz stimulation, 25 ms between flashes), but we systematically varied the interval between A and B, which in the experiment was set to 250 ms. The excitatory neuron receives excitatory input A of synaptic strength (*J_A_* = 0.09) and inhibitory input from the inhibitory neuron (In_I_) of synaptic strength (*J_I_* = 0.04), delayed relative to inhibitory spiking (τ_I_ = 2 ms). The inhibitory neuron receives excitatory input B of synaptic strength (*J_B_* = 0.09). Both neurons decayed to their resting membrane voltage (*V*_m_ = −65 mV) with membrane constant (τ_M_ = 10 ms), consistent with in vivo findings (*90*), and contained a white noise term (In_noise_). They emitted a single spike when they reached the threshold voltage (*V*_T_ = −50 mV), and their voltage was then reset to (*V*_R_ = −70 mV).

The equation of the voltage for each neuron is given by

$$\frac{{\mathrm{dV}}_{E}}{\mathrm{dt}}=\frac{\left(V_{e}-V_{m}\right)+J_{A}{\text{In}}_{A}-J_{I}{\text{In}}_{I}+{\text{In}}_{\text{noise}}}{\tau_{M}}$$

$$\frac{{\mathrm{dV}}_{I}}{\mathrm{dt}}=\frac{\left(V_{I}-V_{m}\right)+J_{B}{\text{In}}_{B}+{\text{In}}_{\text{noise}}}{\tau_{M}}$$

### Statistical analysis

Statistical results (degrees of freedom, *P* values, and statistical values) are reported in figure legends or in table S3. For statistical analysis of neural data, we performed a bootstrap analysis as detailed above. For statistical analysis of behavioral data provided in the manuscript, the Kolmogorov-Smirnov normality test was first performed on the data. If the data failed to meet the normality criterion, statistics relied on nonparametric tests. We therefore represent the median and quartiles of data in boxplots in all figures, in accordance with the use of nonparametric tests. Rank sum and signed rank: We report the signed rank statistic if the number of replicates is too weak to provide the normal *Z* statistic.
