# Supplementary material for: A spatial code for temporal information is necessary for efficient sensory learning
Source: Sci Adv. 2025 Jan 8;11(2):eadr6214. doi: 10.1126/sciadv.adr6214 (PMC11708902; doi:10.1126/sciadv.adr6214)
Supplement: Supplementary file 1 — Supplementary Text Figs. S1 to S6 Tables S1 to S3 [file sciadv.adr6214_sm.pdf]

Science Advances
AAAS

Supplementary Materials for

A spatial code for temporal information is necessary for efficient sensory learning

Sophie Bagur *et al.*

Corresponding author: Brice Bathellier, brice.bathellier@pasteur.fr; Sophie Bagur, sophie.bagur@espci.fr

Sci. Adv. **11**, eadr6214 (2025)
DOI: 10.1126/sciadv.adr6214

This PDF file includes:

Supplementary Text
Figs. S1 to S6
Tables S1 to S3

Supplementary information about the auditory system dataset

To rapidly obtain large datasets from these structures, we used GCAMP6s-based two-photon calcium imaging of either cell bodies (AC and IC, **Fig. 4C,F**) or axonal projections (TH, imaged in AC) (**Fig. 4D**). Collecting data simultaneously from around 1000 AC neurons or TH axonal boutons and from 100 to 200 neurons in IC, we could extensively sample representations in each region. In AC, all 60.822 ROIs were mapped to functional subfields based on tonotopic gradients *(86)* and to the cortical layer from imaging depth (**Fig. S4A-F).** 70% of ROIs were in primary auditory cortex (A1), the largest subfield of AC, but the anterior, suprarhinal and dorsal posterior auditory fields were also covered **(Fig. S4E**). Moreover, with recording depth reaching up to 600 µm, we sampled neurons from layers I to V with an emphasis on layers II and III (**Fig. S4F**). Therefore, with the exception of layer VI and of the small ventro-posterior subfield, the whole of primary and secondary AC was extensively covered. Inputs from TH were sampled with 39.191 putative TH axonal boutons spread across AC (75% of ROIs in A1) (**Fig. 3D**) and validated post-hoc with the thalamic marker VGLUt2 (**Fig. S4G,H**). In addition, we recorded 15.132 ROIs in the dorsal IC down to 250µm depth (**Fig. 4F**).

Since calcium imaging and deconvolution has not been verified for TH axons, we performed electrophysiological recording in primary and secondary auditory TH (498 single units, **Fig. 3E**). Electrophysiology was also used to cover the central inferior colliculus (563 single units), the main primary subregion of this structure (**Fig. 3G**). Electrode locations were identified with post-hoc histology and short-latency responses (**Fig. 3E,G**).

Calcium signals were temporally deconvolved using a linear algorithm to retrieve estimates of neuronal firing rate variations that are robust to parameterization errors. This allowed us to reach a ~150 ms temporal precision as estimated from responses to amplitude modulated sounds (**Fig. 3C,D,F panel v**). The temporal modulations of our sounds were chosen to evolve at timescales compatible with this resolution of calcium imaging. This was confirmed by our decomposition of neural population activity into specific timescales using Fourier analysis (**Fig. 4E**). This revealed that even with electrophysiology, in which activity contained information at fast timescales up to 30Hz, information nonetheless saturated at around 3Hz. Therefore, all information needed to discriminate our sounds is available below 3Hz, which matches calcium imaging resolution, with information at faster timescales being redundant.

Supplemental mathematical derivations

Population vector notation

For N neurons indexed n, S sounds indexed s, T time steps indexed t and R sound repeats indexed r, population activity is written:

$$\vec{\nu}_{s,r,t} = (\nu_{1,s,r,t}; ...; \nu_{N,s,r,t})$$

and corresponds to the vector of instantaneous neuronal firing rates for condition (s, r, t). For more compact notations, time averaging is written implicitly:

$$\vec{\nu}_{s,r} = \frac{1}{T} \sum_t \vec{\nu}_{s,r,t}$$

The scalar product of two population vectors is defined as:

$$\vec{\nu}_{s,r,t}.\vec{\nu}_{s,r',t} = \sum_n \nu_{n,s,r,t}\nu_{n,s,r',t}$$

Estimate of noise-free correlation

If noise is additive, we can decompose a single-trial population vector for sound s $\vec{\nu}_{s,r}$ as the sum of a noise-free vector $\vec{\nu}_s$ and of an additive normally distributed noise vector $\vec{\xi}_{s,r}$ of mean 0 and variance σ^2/N. Noise is uncorrelated across trials and sounds. Therefore, in the limit of a large number N of neurons, the scalar product between noise vectors of two different trials r and r' or sounds s and s' converges to zero (i.e. $\vec{\xi}_{s,r}.\vec{\xi}_{s',r'} = \sigma^2\delta_{rr'}\delta_{ss'}$ where $\delta_{rr'}$ is the Kronecker symbol).

For compactness of the demonstration, we assume here that population vectors have zero mean along the neuronal dimension, but the result holds for non-zero mean. The correlation coefficient between two noise-free vectors $\vec{\nu}_s$ and $\vec{\nu}_{s'}$ of mean 0 can be written as:

$$\rho_{\vec{\nu}_s\vec{\nu}_{s'}} = \frac{\vec{\nu}_s.\vec{\nu}_{s'}}{\sqrt{\vec{\nu}_s^{\,2}\vec{\nu}_{s'}^{\,2}}}$$

With trial noise, this becomes:

$$\rho_{\vec{\nu_{s,r}}\vec{\nu_{s',r}}} = \frac{\left(\vec{\nu_s} + \vec{\xi_r}\right)\left(\vec{\nu_{s'}} + \vec{\xi_r'}\right)}{\sqrt{\left(\vec{\nu_s} + \vec{\xi_r}\right)^2\left(\vec{\nu_{s'}} + \vec{\xi_r'}\right)^2}}$$

Because $\vec{\xi_r}$ and $\vec{\xi_r'}$ are two uncorrelated noise vectors, we can write in the limit of large N:

$$\rho_{\vec{\nu}_{s,r}\vec{\nu}_{s',r}} = \frac{\vec{\nu}_s.\vec{\nu}_{s'}}{\sqrt{\vec{\nu}_s^{\,2}\vec{\nu}_{s'}^{\,2}}} \frac{1}{\sqrt{(1 + \frac{N\sigma^2}{\vec{\nu}_s^{\,2}})(1 + \frac{N\sigma^2}{\vec{\nu}_{s'}^{\,2}})}}$$

which can be rewritten as :

$$\rho_{\vec{\nu}_{s,r}\vec{\nu}_{s',r}} = \frac{\rho_{\vec{\nu}_s\vec{\nu}_{s'}}}{\sqrt{(1+\frac{N\sigma^2}{\vec{\nu_s}^2})(1+\frac{N\sigma^2}{\vec{\nu_{s'}}^2})}}$$

If one wants to compare correlation coefficient from different datasets with different unknown σ's, this correction factor may introduce discrepancies that are only related to the noise magnitudes.

However, because $\rho_{\vec{\nu}_s\vec{\nu}_s} = 1$ one can derives from the previous equation that:

$$\rho_{\vec{\nu}_{s,r}\vec{\nu}_{s,r'}} = \frac{1}{(1+\frac{N\sigma^2}{\vec{\nu_s}^2})}$$

Combining the last two equations yields in the limit of large N:

$$\rho_{\vec{\nu}_s\vec{\nu_{s'}}} \approx \frac{\rho_{\vec{\nu}_{s,r}\vec{\nu}_{s',r'}}}{\sqrt{\rho_{\vec{\nu}_{s,r}\vec{\nu}_{s,r'}}\rho_{\vec{\nu}_{s',r}\vec{\nu}_{s',r'}}}}$$

Note that the formula here is shown only for pairs of trials. In practice, for a finite N, the estimate of the correlation coefficient can be improved by averaging across multiple trial pairs using the formula:

$$\rho_{\vec{\nu}_s\vec{\nu}_{s'}} \approx \frac{\frac{1}{R^2}\sum_{r,r'}\rho_{\vec{\nu}_{s,r}\vec{\nu}_{s',r'}}}{\sqrt{\frac{1}{R^2(1-R)^2}\left(\sum_{r\neq r'}\rho_{\vec{\nu}_{s,r}\vec{\nu}_{s,r'}}\right)\left(\sum_{r\neq r'}\rho_{\vec{\nu}_{s',r}\vec{\nu}_{s',r'}}\right)}}$$

Supplementary Figures and Tables

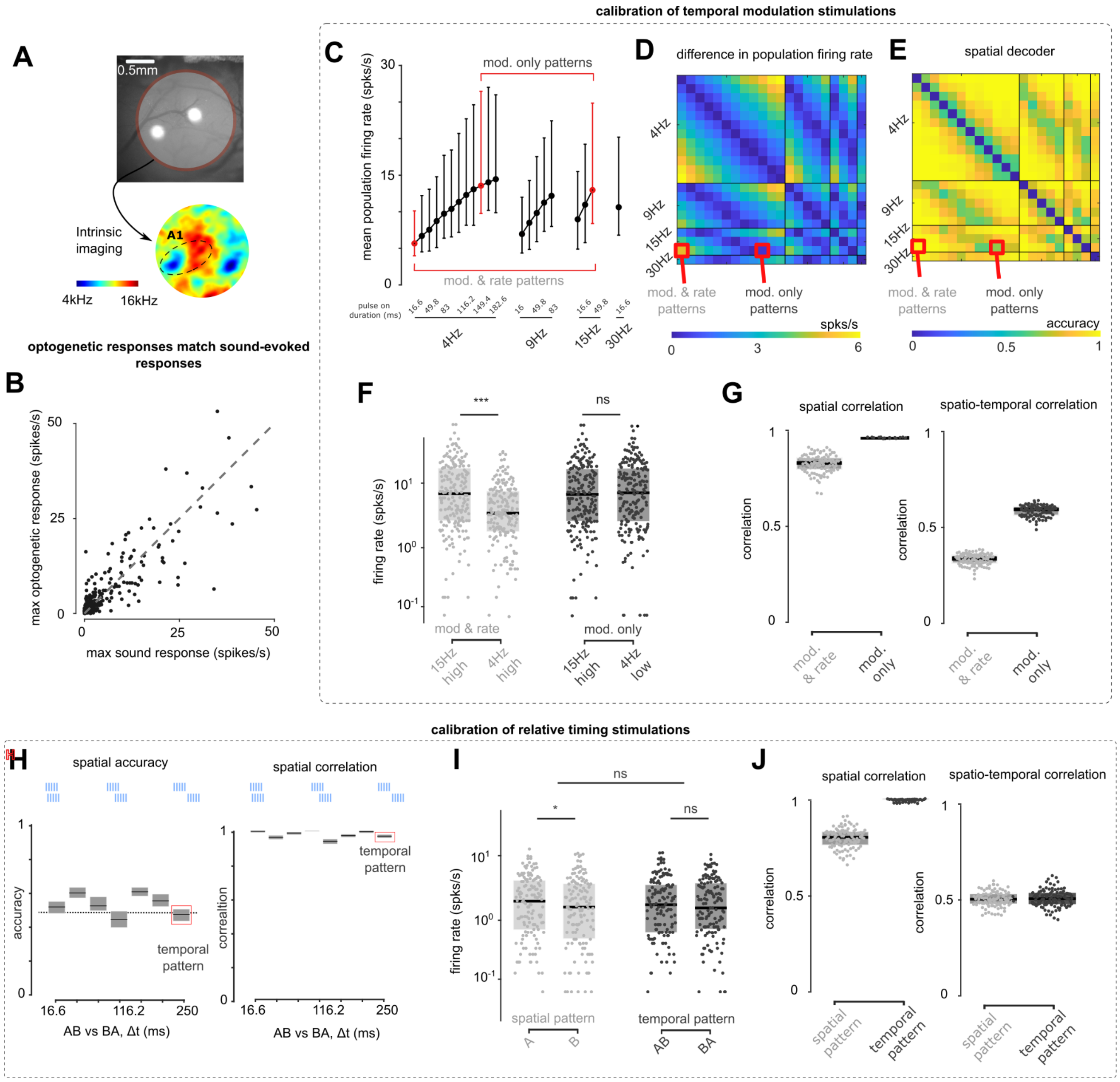

Fig. S1. Parameter search to generate optogenetic stimulations

A. Example mouse showing two optogenetic spots placed at opposite ends of the A1 tonotopic gradient as established using intrinsic imaging (bottom). **B**. Mean firing rate during the optogenetic stimulation and sound stimulus that evoked the highest firing rate in each neuron (n=344 units). **C-G** : All results concern the temporal modulation task. **C**. Global population firing rate in response to stimulations of different frequencies and with varying pulse width. Pulse width can thus be used to compensate for the firing rate differences induced by the varying number of pulses associated with each frequency. (mean +/- quartiles) **D-E.** For all pairs of stimulations parameters, difference in global firing rate (**D**) and accuracy of a spatial decoder trained to discriminate the two (**E**). The pairs used for the temporal pattern (matched firing rates, low spatial accuracy) and the spatial pattern (different firing rates, high spatial accuracy) are highlighted in red. **F.** Average firing rates during each stimulation showing that firing rates are equal for the temporal stimulation pair and

unequal for the spatial stimulation (paired Wilcoxon test, p = 3.4e-24, signed rank value = 16774, 7711, n=344). **G.** Spatial and spatio-temporal correlation between neural activity in response to stimulation pairs (n=344 units, bootstrap over units). **H-J** : All results concern the relative timing task. **H.** Spatial accuracy (left) and spatial correlation (right) between AB vs BA pairs with varying delays. We tested these varying delays to verify that they provided low levels of spatial information and found that it dropped to almost chance levels when A and B were separated by a 25ms delay, however spatial correlation was high for all delays indicating that this information, although present, is likely hard to exploit. **I.** Accuracy of the spatial decoder and spatial correlation on pairs depending on the separation between A and B (n=344 units, bootstrap over units, p-value of accuracy vs chance level of 0.5: 0.01, 0.01, 0.01, 0.05, 0.43). **I.** Average firing rates during each stimulation showing that firing rates are equal for the temporal stimulation pair and show a small but significant difference for spatial stimulation. Given that the low frequency region spot (here called A) was situated closer to the electrode, this difference may be due to sampling bias. (paired Wilcoxon test, p = 0.02, 0.21, signed rank value = 5500, 5230, n=344). There was no overall difference in firing rates between the patterns used in the spatial vs temporal protocoles (paired Wilcoxon test, p = 0.97, signed rank value = 9929, n=344). **J.** Spatial and spatio-temporal correlation between neural activity in response to stimulation pairs (n=344 units, bootstrap over units).

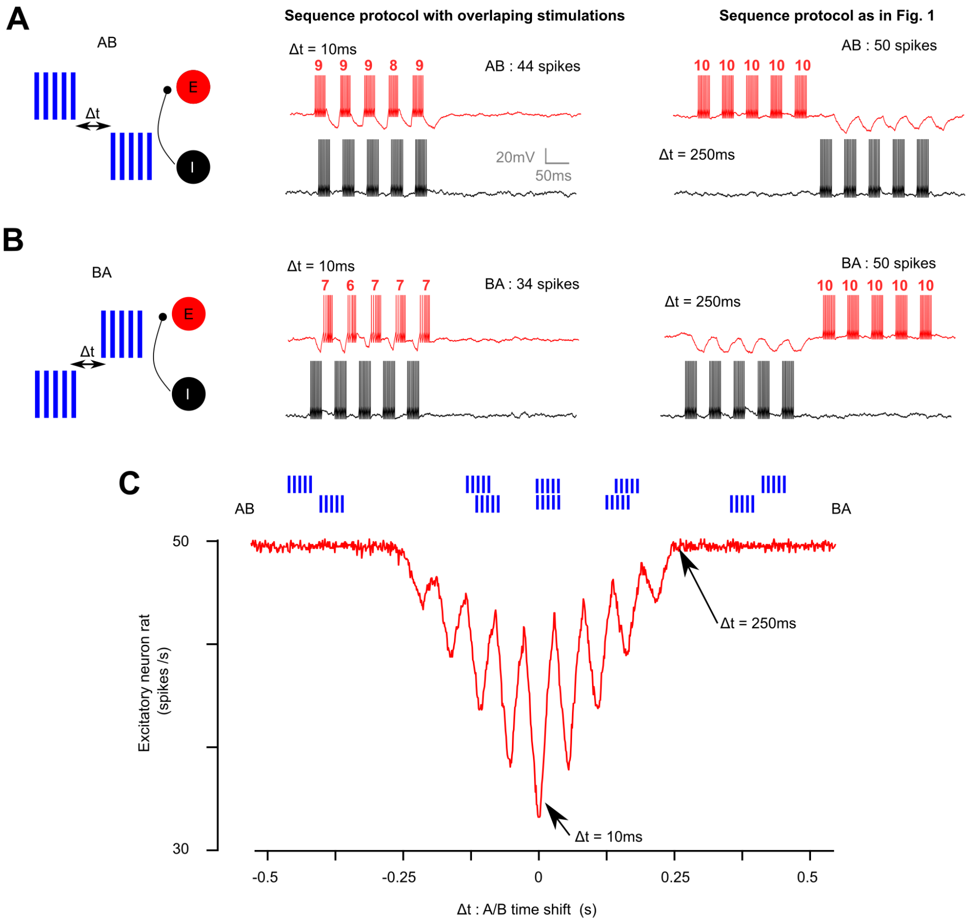

Fig. S2. Synaptic integration converts temporal information into different firing rates providing spatial information at short time scales.

Neurons integrate synaptic inputs over the timescale of their membrane time constant. Hence the number of output spikes depends not only on the number and size of synaptic inputs but also on their relative timing. This property is reinforced by synaptic connectivity such that simple circuits can detect time shifts between incoming inputs and thereby transform temporal information into specific firing rates for different neurons which generates spatial information. **A.B** To illustrate this point and evaluate under which conditions temporal information injected in the AC may give rise to salient spatial representations, we simulated the activity of an integrate-and-fire neuron E receiving inhibitory input from a neuron I. The inputs received by E and I are trains of 5 current pulses at 20Hz (pulse duration 25ms, total duration 225ms) simulating ChR2 activations by light as in **Fig. 1**. The timing of the inputs to neurons E and I are shifted by a time Δt to generate temporal information. Exemple simulations indicate that when the time shift is small and the inputs to E and I overlap in time (middle panels), neuron E emits fewer action potentials than when the inputs are well separated in time (right panels). **C.** Quantification of the effect exemplified in panels **A** and **B**, showing the firing rate of neuron E for a range of time shifts Δt. For |Δt| < 250ms, when the two inputs are separated by less than 25ms, neuron E emits fewer action potentials than for |Δt| > 250ms and the number of action potentials depends on Δt. Hence, temporal information is converted partially to rate information for |Δt| < 250ms but not for |Δt| > 250ms. The boundary value for |Δt| (here ~250ms) depends on the membrane time constant of the neurons (here set to 10ms, similar to values reported *in vivo*, *(90)*). In general, this simulation indicates that in order to avoid the

conversion of temporal information into rate information, the temporal sequences injected in cortex must avoid temporal contiguities over the time scale of the membrane time constant. Moreover, the use of time-reversed sequences tends to ensure smaller firing rate differences across sequences, compared to time sequences that are not symmetric of each other.

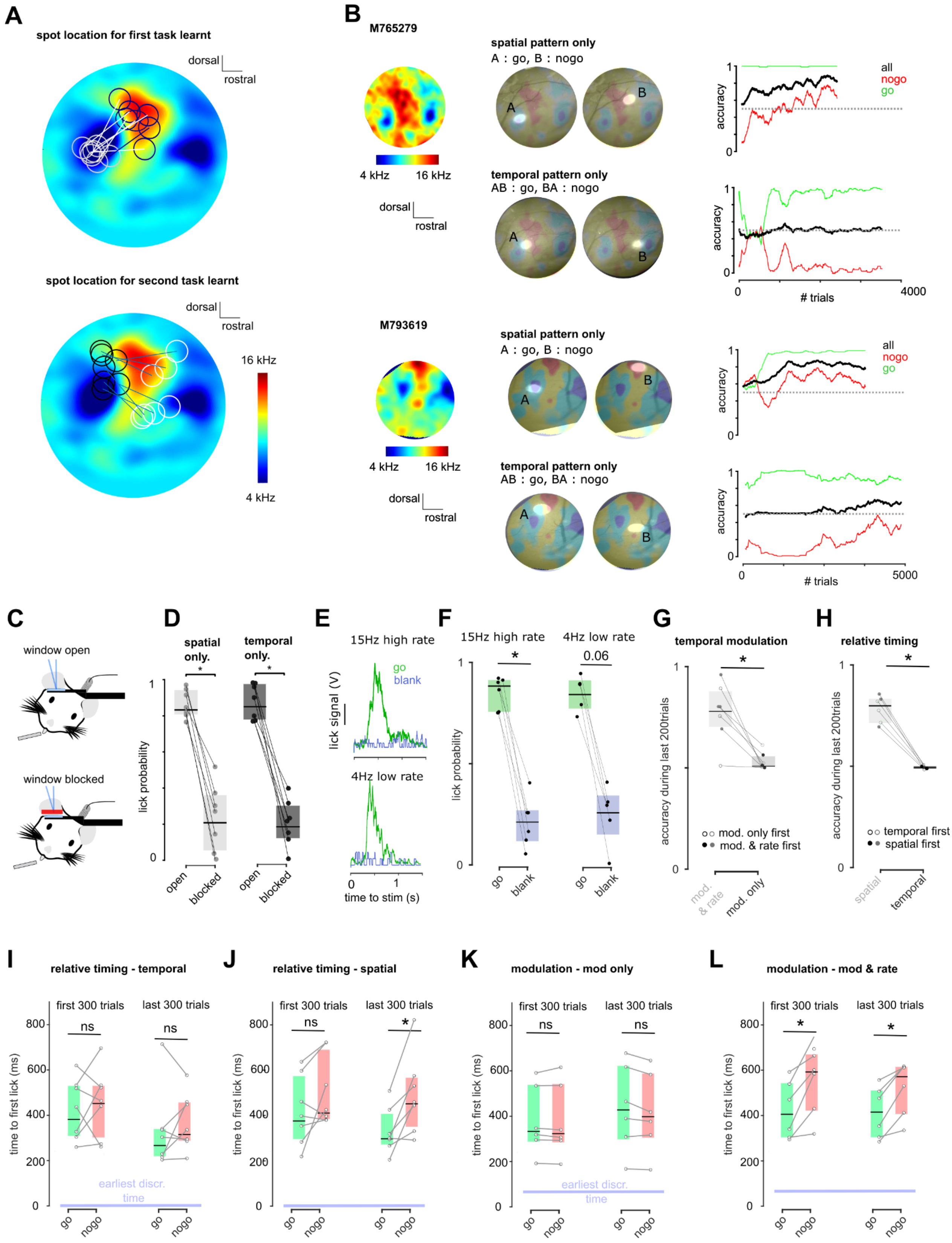

Fig. S3. Details of behavioral learning in optogenetic cortical stimulation protocol.
A. Population average intrinsic imaging map of tonotopic areas in AC showing the localization of all spots used for optogenetic stimulation (n=7 mice). **B.** Intrinsic maps and spots used for

stimulation with learning curves from two example mice in both tasks. **C-D.** Control experiment showing that response to optogenetic stimulation is specific to cortical activation: mice ceased responding to light stimulation when the cranial window was blocked by a small cache that left all other light cues intact. Note also that the lick probability for temporal and rate patterns is identical during this initial phase. (paired Wilcoxon test, p = 0.0156, signed rank value = 28, n=7). **E-F** Control experiment showing that mice respond equally well to the 15Hz high firing rate and the 4Hz low firing rate stimulations. Responses are compared to control trials in which no light is presented to ensure that licking is specific to the stimulation. **E.** Average lick trace in response to the 15Hz and 4Hz stimulations vs blank control stimulations for two example mice. **F.** Lick probability to the 15Hz and 4Hz stimulations vs blank control stimulations. (paired Wilcoxon test, p = 0.0313, 0.0625 signed rank value = 21,15, n=6). **G-H.** Accuracy over the last 300 trials for all mice in both tasks. (paired Wilcoxon test, G : p = 0.015, signed rank value = 28, n=7, H : p = 0.0313, signed rank value = 21, n=6). **I-L.** Average time to first lick for the four optogenetic tasks on Go and NoGo trials. For each task the average over the first and last 300 trials of training are shown. The blue line indicates the earliest time at which the Go and NoGo stimulations diverge and so only animals responding after have time to integrate the temporal structure.

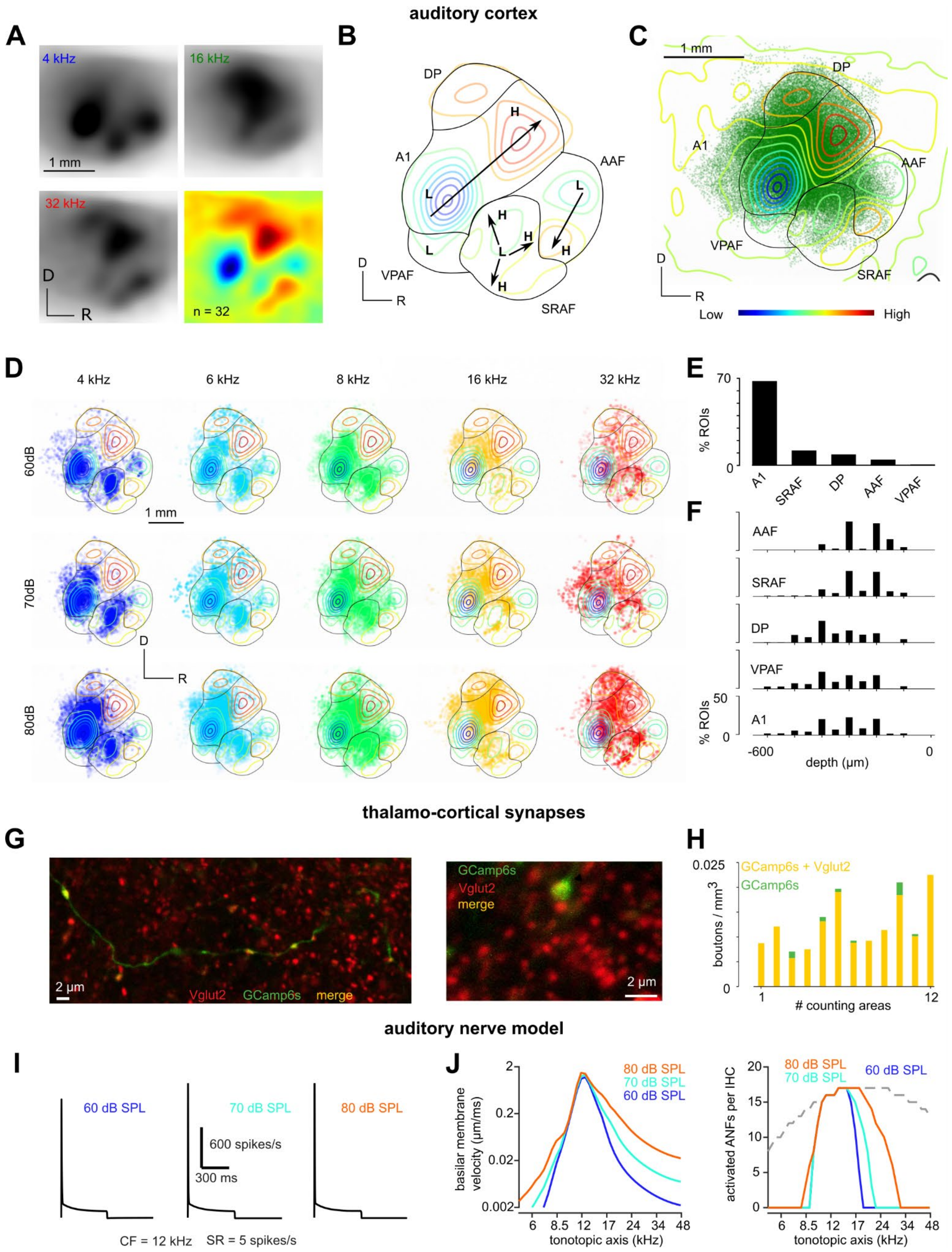

Fig. S4. Details of auditory system sampling.

A. Mean intrinsic imaging responses (n=32 mice) for 4, 16 and 32 kHz sounds (black) and the subtraction of 32kHz and 4kHz maps (colour). This extended data set allowed us to construct a consensus map to align mice included in the study. **B.** Illustration of method used to identify

AC subregions based on the tonotopic gradients established in *(86)*. **C.** Localization of all recorded ROIs on the consensus tonotopic map with AC subregions. **D.** Localization of responsive neurons to increasing frequency and intensity. Note the larger recruitment with stronger intensity and the spatial shift with frequency. **E.** Proportion of units per subarea. **F.** Depth distribution of units per subarea. **G**. Example thalamocortical axon expressing GCaMP6s merged with Vglut2. Thalamic axonal boutons expressing Vglut2 appear yellow as shown in the magnified region (right). **H.** Density of labeled boutons (Vglut2^{+};GCaMP6s-expressing in yellow; GCaMP6s alone in green) in layer 1 of the AC (12 sample regions; 4 regions per confocal image; means and STD: 0.0122±0.0052, 0.0005±0.0008, density of co-labelled and green only boutons, respectively). **I**. Peristimulus time histogram of an auditory nerve fiber (ANF) with a characteristic frequency equal to that of the presented 12-kHz tone burst (10-ms rise/fall, 500-ms duration) with increasing level from 60, 70 and 80 dB SPL. Note the rapid adaptation of the firing. **J**. Basilar membrane velocity and sound-activated auditory nerve fibers per inner hair cell (IHC) along the tonotopic axis. Note the reduced frequency selectivity with the increasing intensity. Gray dashed line shows the mouse synaptic cochleogram. The criterion for sound-activated auditory nerve fibers was 10 spikes/s above the spontaneous rate.

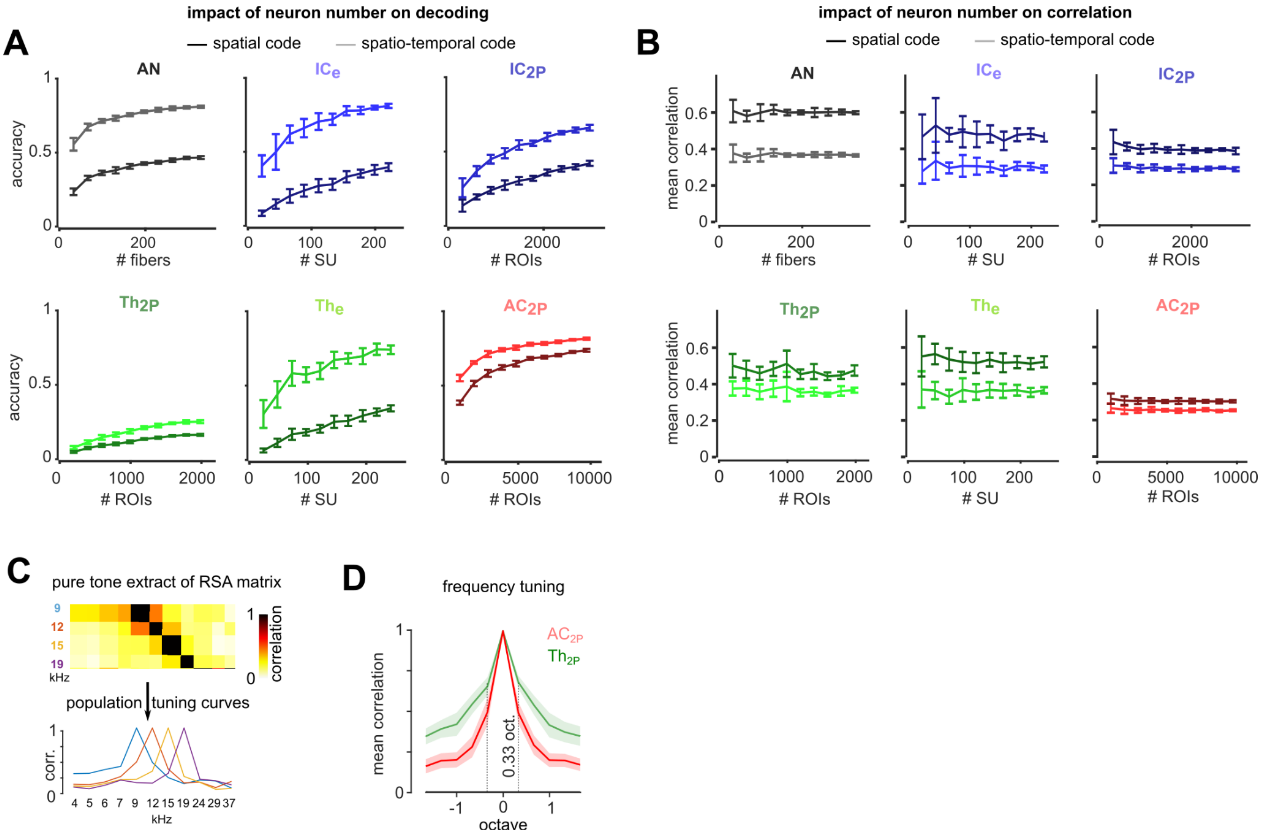

Fig. S5. Details and controls of population and single-cell level encoding

A. Decoding accuracy for spatio-temporal and spatial codes in each area with varying numbers of sub-selected neurons. **B.** Noise-corrected correlation for spatio-temporal and spatial code in each area with varying numbers of sub-selected neurons. **C.** Illustration of method to calculate population tuning curves shown in D from RSA matrix. **D.** Mean noise-corrected correlation between pure tones as a function of their frequency separation.

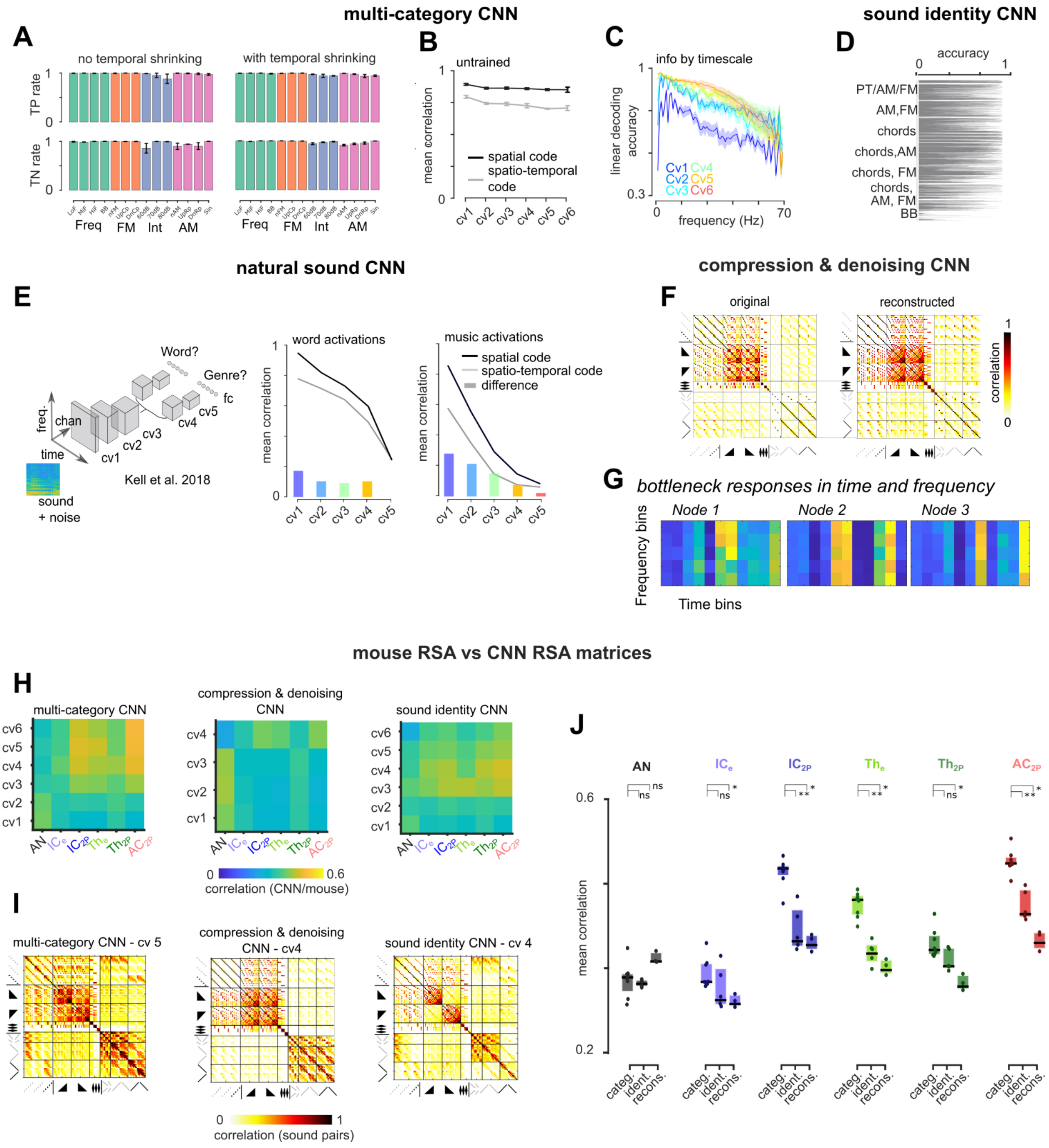

Fig. S6. CNN performance and detailed comparison with neural data.
A. Category by category performance of CNNs trained without shrinking of the temporal dimension (left) or with (right) (n=8, error bars are sem). **B.** Mean response correlations from RSA matrices from untrained networks with the same architecture as those trained on the multi-category task (n=8, error bars are sem). **C.** Mean decoding accuracy based on successive Fourier coefficients of CNN responses. 0Hz = spatial code (n=8, shaded areas are sem) **D.** Accuracy for each sound for the CNNs trained to identify all 2169 sounds individually. **E.** Mean correlations from the network trained on natural sounds from Kell et al *(60)* for musical snippets (left) or words (center). **F.** Representation Similarity Analysis matrix of original sounds and reconstructed sounds showing that the autoencoder fully preserved the relations

between all the sounds. **G.** Responses from 3 example nodes in the autoencoder bottleneck to a set of short pure tones covering all frequency and time bins. This shows that nodes respond to multiple time bins, including well separated timebins (ex node 2 and 3) and that this response also depends on the frequency (ex node 1). This illustrates that the autoencoder does not possess an explicit temporal dimension but instead integrates information over time in this layer. **H.** Average correlation between the RSA matrices from each region of the mouse auditory system and the different layers of networks performing different tasks. This shows that early stages of the auditory system differed strongly from all layers of the CNNs and that IC, TH and AC reassembled later CNN layers but without any clear indication of hierarchical organization. **I.** Spatial coded RSA matrix from the closest resembling layer of CNNs performing different tasks to the AC. **J.** Correlation between the RSA matrices from each region of the mouse auditory system and the closest resembling layer of CNNs performing different tasks. Each point represents one network trained on the task either with different architecture or different random initialization. (Statistics are sign rank tests, n=8,8,4, p-values in **Table S3**)

Table S1. Details of dataset

Brain region	*Recording method*	*Units recorded*	*Responsive units*	*Animal number*	*Session number*	*Units per animal (min, mean, max)*	*Units per session (min, mean, max)*
Auditory cortex	Cell body 2 photon calcium imaging	60822	19414 (32%)	7	60	2164 / 8688 / 20631	57 / 1013 / 1782
Auditory thalamus	Axonal bouton 2 photon calcium imaging	39191	3969 (12%)	4	24	1280 / 9287 / 19870	477 / 1632 / 3120
	Single unit electrophysiology	498	484 (97%)	10	33	4 / 49 / 113	2 / 15 / 32
Inferior colliculus	Cell body 2 photon calcium imaging	15312	5936 (39%)	30	101	25 / 510 / 2975	25 / 151 / 495
	Single unit electrophysiology	563	442 (78%)	11	30	10 / 56 / 119	4 / 18 / 54

Table S2. Sound parameters

			Start freq. (kHz)	*Stop freq. (kHz)*	*Start int. (dB)*	*Stop int. (dB)*	*Dur. (ms)*
1		*blank*	*NaN*	*NaN*	*NaN*	*NaN*	*500*
2	*Pure tones*	*tono60dB_4kHz*	*4*	*4*	*60*	*60*	*500*
3		*tono60dB_5kHz*	*5*	*5*	*60*	*60*	*500*
4		*tono60dB_6kHz*	*6*	*6*	*60*	*60*	*500*
5		*tono60dB_7kHz*	*7*	*7*	*60*	*60*	*500*
6		*tono60dB_9kHz*	*9*	*9*	*60*	*60*	*500*
7		*tono60dB_12kHz*	*12*	*12*	*60*	*60*	*500*
8		*tono60dB_15kHz*	*15*	*15*	*60*	*60*	*500*
9		*tono60dB_19kHz*	*19*	*19*	*60*	*60*	*500*
10		*tono60dB_24kHz*	*24*	*24*	*60*	*60*	*500*
11		*tono60dB_29kHz*	*29*	*29*	*60*	*60*	*500*
12		*tono60dB_37kHz*	*37*	*37*	*60*	*60*	*500*
13		*tono70dB_4kHz*	*4*	*4*	*70*	*70*	*500*
14		*tono70dB_5kHz*	*5*	*5*	*70*	*70*	*500*
15		*tono70dB_6kHz*	*6*	*6*	*70*	*70*	*500*
16		*tono70dB_7kHz*	*7*	*7*	*70*	*70*	*500*
17		*tono70dB_9kHz*	*9*	*9*	*70*	*70*	*500*
18		*tono70dB_12kHz*	*12*	*12*	*70*	*70*	*500*
19		*tono70dB_15kHz*	*15*	*15*	*70*	*70*	*500*
20		*tono70dB_19kHz*	*19*	*19*	*70*	*70*	*500*
21		*tono70dB_24kHz*	*24*	*24*	*70*	*70*	*500*
22		*tono70dB_29kHz*	*29*	*29*	*70*	*70*	*500*
23		*tono70dB_37kHz*	*37*	*37*	*70*	*70*	*500*
24		*tono80dB_4kHz*	*4*	*4*	*80*	*80*	*500*
25		*tono80dB_5kHz*	*5*	*5*	*80*	*80*	*500*
26		*tono80dB_6kHz*	*6*	*6*	*80*	*80*	*500*
27		*tono80dB_7kHz*	*7*	*7*	*80*	*80*	*500*
28		*tono80dB_9kHz*	*9*	*9*	*80*	*80*	*500*
29		*tono80dB_12kHz*	*12*	*12*	*80*	*80*	*500*
30		*tono80dB_15kHz*	*15*	*15*	*80*	*80*	*500*
31		*tono80dB_19kHz*	*19*	*19*	*80*	*80*	*500*
32		*tono80dB_24kHz*	*24*	*24*	*80*	*80*	*500*
33		*tono80dB_29kHz*	*29*	*29*	*80*	*80*	*500*
34		*tono80dB_37kHz*	*37*	*37*	*80*	*80*	*500*
35	*Pure up ramps*	*Up4kHz*	*4*	*4*	*60*	*80*	*500*
36		*Up6kHz*	*6*	*6*	*60*	*80*	*500*
37		*Up9kHz*	*9*	*9*	*60*	*80*	*500*
38		*Up15kHz*	*15*	*15*	*60*	*80*	*500*
39		*Up24kHz*	*24*	*24*	*60*	*80*	*500*
40	*Chord up ramps*	*Up4+6kHz*	*4, 6*	*4, 6*	*60*	*80*	*500*
41		*Up4+9kHz*	*4, 9*	*4, 9*	*60*	*80*	*500*
42		*Up4+15kHz*	*4, 15*	*4, 15*	*60*	*80*	*500*
43		*Up4+24kHz*	*4, 24*	*4, 24*	*60*	*80*	*500*
44		*Up6+9kHz*	*6, 9*	*6, 9*	*60*	*80*	*500*
45		*Up6+15kHz*	*6, 15*	*6, 15*	*60*	*80*	*500*
46		*Up6+24kHz*	*6, 24*	*6, 24*	*60*	*80*	*500*
47		*Up9+15kHz*	*9, 15*	*9, 15*	*60*	*80*	*500*
48		*Up9+24kHz*	*9, 24*	*9, 24*	*60*	*80*	*500*
49		*Up15+24kHz*	*15, 24*	*15, 24*	*60*	*80*	*500*
50		*Up4+6+9+15kHz*	*4, 6, 9, 15*	*4, 6, 9, 15*	*60*	*80*	*500*

51		Up4+6+9+24kHz	4, 6, 9, 24	4, 6, 9, 24	60	80	500
52		Up4+6+15+24kHz	4, 6, 15, 24	4, 6, 15, 24	60	80	500
53		Up4+9+15+24kHz	4, 9, 15, 24	4, 9, 15, 24	60	80	500
54		Up6+9+15+24kHz	6, 9, 15, 24	6, 9, 15, 24	60	80	500
55		UpmultiHz	4, 6, 9, 15, 24	4, 6, 9, 15, 24	60	80	500
56	Pure down ramps	Down4kHz	4	4	80	60	500
57		Down6kHz	6	6	80	60	500
58		Down9kHz	9	9	80	60	500
59		Down15kHz	15	15	80	60	500
60		Down24kHz	24	24	80	60	500
61	Chord down ramps	Down4+6kHz	4, 6	4, 6	80	60	500
62		Down4+9kHz	4, 9	4, 9	80	60	500
63		Down4+15kHz	4, 15	4, 15	80	60	500
64		Down4+24kHz	4, 24	4, 24	80	60	500
65		Down6+9kHz	6, 9	6, 9	80	60	500
66		Down6+15kHz	6, 15	6, 15	80	60	500
67		Down6+24kHz	6, 24	6, 24	80	60	500
68		Down9+15kHz	9, 15	9, 15	80	60	500
69		Down9+24kHz	9, 24	9, 24	80	60	500
70		Down15+24kHz	15, 24	15, 24	80	60	500
71		Down4+6+9+15kHz	4, 6, 9, 15	4, 6, 9, 15	80	60	500
72		Down4+6+9+24kHz	4, 6, 9, 24	4, 6, 9, 24	80	60	500
73		Down4+6+15+24kHz	4, 6, 15, 24	4, 6, 15, 24	80	60	500
74		Down4+9+15+24kHz	4, 9, 15, 24	4, 9, 15, 24	80	60	500
75		Down6+9+15+24kHz	6, 9, 15, 24	6, 9, 15, 24	80	60	500
76		DownmultiHz	4, 6, 9, 15, 24	4, 6, 9, 15, 24	80	60	500
77	Sinusoid AM modulation	Sin1Hz9kHz	9	9	60 - 80	60 - 80	1000
78		Sin3Hz9kHz	9	9	60 - 80	60 - 80	1000
79		Sin7Hz9kHz	9	9	60 - 80	60 - 80	1000
80		Sin20Hz9kHz	9	9	60 - 80	60 - 80	1000
81		Sin1Hz24kHz	24	24	60 - 80	60 - 80	1000
82		Sin3Hz24kHz	24	24	60 - 80	60 - 80	1000
83		Sin7Hz24kHz	24	24	60 - 80	60 - 80	1000
84		Sin20Hz24kHz	24	24	60 - 80	60 - 80	1000
85		Sin1HzWhitenoise	WN	WN	60 - 80	60 - 80	1000
86		Sin3HzWhitenoise	WN	WN	60 - 80	60 - 80	1000
87		Sin7HzWhitenoise	WN	WN	60 - 80	60 - 80	1000
88		Sin20HzWhitenoise	WN	WN	60 - 80	60 - 80	1000
89	Up chirp varying speed	ChirpUp4kHz60dB100ms	4	9	60	60	100
90		ChirpUp4kHz60dB250ms	4	9	60	60	250
91		ChirpUp4kHz60dB500ms	4	9	60	60	500
92		ChirpUp24kHz60dB100ms	9	24	60	60	100
93		ChirpUp24kHz60dB250ms	9	24	60	60	250
94		ChirpUp24kHz60dB500ms	9	24	60	60	500
95	Down chirp varying speed	ChirpDown4kHz60dB100ms	9	4	60	60	100
96		ChirpDown4kHz60dB250ms	9	4	60	60	250
97		ChirpDown4kHz60dB500ms	9	4	60	60	500
98		ChirpDown24kHz60dB100ms	24	9	60	60	100
99		ChirpDown24kHz60dB250ms	24	9	60	60	250
100		ChirpDown24kHz60dB500ms	24	9	60	60	500
101		ChirpUpclose4to6kHz60dB	4	6	60	60	500

102	Up chirp - 60 dB	*ChirpUpclose4to9kHz60dB*	4	9	60	60	500
103		*ChirpUpclose4to15kHz60dB*	4	15	60	60	500
104		*ChirpUpclose4to24kHz60dB*	4	24	60	60	500
105		*ChirpUpclose6to9kHz60dB*	6	9	60	60	500
106		*ChirpUpclose6to15kHz60dB*	6	15	60	60	500
107		*ChirpUpclose6to24kHz60dB*	6	24	60	60	500
108		*ChirpUpclose9to15kHz60dB*	9	15	60	60	500
109		*ChirpUpclose9to24kHz60dB*	9	24	60	60	500
110		*ChirpUpclose15to24kHz60dB*	15	24	60	60	500
111	Down chirp - 60 dB	*ChirpDownclose6to4kHz60dB*	6	4	60	60	500
112		*ChirpDownclose9to4kHz60dB*	9	4	60	60	500
113		*ChirpDownclose15to4kHz60dB*	15	4	60	60	500
114		*ChirpDownclose24to4kHz60dB*	24	4	60	60	500
115		*ChirpDownclose9to6kHz60dB*	9	6	60	60	500
116		*ChirpDownclose15to6kHz60dB*	15	6	60	60	500
117		*ChirpDownclose24to6kHz60dB*	24	6	60	60	500
118		*ChirpDownclose15to9kHz60dB*	15	9	60	60	500
119		*ChirpDownclose24to9kHz60dB*	24	9	60	60	500
120		*ChirpDownclose24to15kHz60dB*	24	15	60	60	500
121	Up chirp - 80 dB	*ChirpUpclose4to6kHz80dB*	4	6	80	80	500
122		*ChirpUpclose4to9kHz80dB*	4	9	80	80	500
123		*ChirpUpclose4to15kHz80dB*	4	15	80	80	500
124		*ChirpUpclose4to24kHz80dB*	4	24	80	80	500
125		*ChirpUpclose6to9kHz80dB*	6	9	80	80	500
126		*ChirpUpclose6to15kHz80dB*	6	15	80	80	500
127		*ChirpUpclose6to24kHz80dB*	6	24	80	80	500
128		*ChirpUpclose9to24kHz80dB*	9	15	80	80	500
129		*ChirpUpclose9to24kHz80dB*	9	24	80	80	500
130		*ChirpUpclose15to24kHz80dB*	15	24	80	80	500
131	Down chirp - 80 dB	*ChirpDownclose6to4kHz80dB*	6	4	80	80	500
132		*ChirpDownclose9to4kHz80dB*	9	4	80	80	500
133		*ChirpDownclose15to4kHz80dB*	15	4	80	80	500
134		*ChirpDownclose24to4kHz80dB*	24	4	80	80	500
135		*ChirpDownclose9to6kHz80dB*	9	6	80	80	500
136		*ChirpDownclose15to6kHz80dB*	15	6	80	80	500
137		*ChirpDownclose24to6kHz80dB*	24	6	80	80	500
138		*ChirpDownclose15to9kHz80dB*	15	9	80	80	500
139		*ChirpDownclose24to9kHz80dB*	24	9	80	80	500
140		*ChirpDownclose24to15kHz80dB*	24	15	80	80	500

Table S3. Detail of statistical comparisons

Fig. 4D. Bootstrap comparison of difference between temporal and rate accuracy in structure X vs in AC						
	AN	ICE	IC	THE	TH	
(Temp-Rate) norm	<0.01	<0.01	0.01	0.04	0.15	
Fig. 5D Bootstrap comparison of temporal and rate mean correlations in structure X vs in AC						
	AN	ICE	IC	THE	TH	
Temporal	<0.01	0.43	0.12	0.01	<0.01	
Rate	<0.01	<0.01	<0.01	<0.01	<0.01	
(Temp-Rate) norm	<0.01	<0.01	0.032	0.01	0.086	
Fig. 5E. Bootstrap comparison of RSA matrix similarity in structure X vs in AC						
	AN	ICE	IC	THE	TH	
Temp vs Rate	<0.01	<0.01	0.01	<0.01	0.01	
Fig. 5H-K. Bootstrap comparison of rate mean correlations in structure X vs in AC						
	AN	ICE	IC	THE	TH	
5F - freq PT	<0.01	0.25	0.61	<0.01	0.27	
5G - FM direction	<0.01	<0.01	<0.01	<0.01	0.04	
5H - AM direction	<0.01	<0.01	<0.01	<0.01	0.1	
Fig. 6. Bootstrap comparison of learning rates with TH vs AC representations						
6K - freq	0.31					
6L - FM direction	<0.01					
